# Bifunctional Tagging through N-Doped Ozonide for Charge Switching and Isomeric Characterization of Glycerophospholipids Using Tandem Mass Spectrometry

**DOI:** 10.1021/acs.analchem.5c00443

**Published:** 2025-04-17

**Authors:** Chia-Lung Tsai, Xi Chen, Ramidi G. Reddy, Xin Yan

**Affiliations:** Department of Chemistry, Texas A&M University, 580 Ross St., College Station, Texas 77843, United States

## Abstract

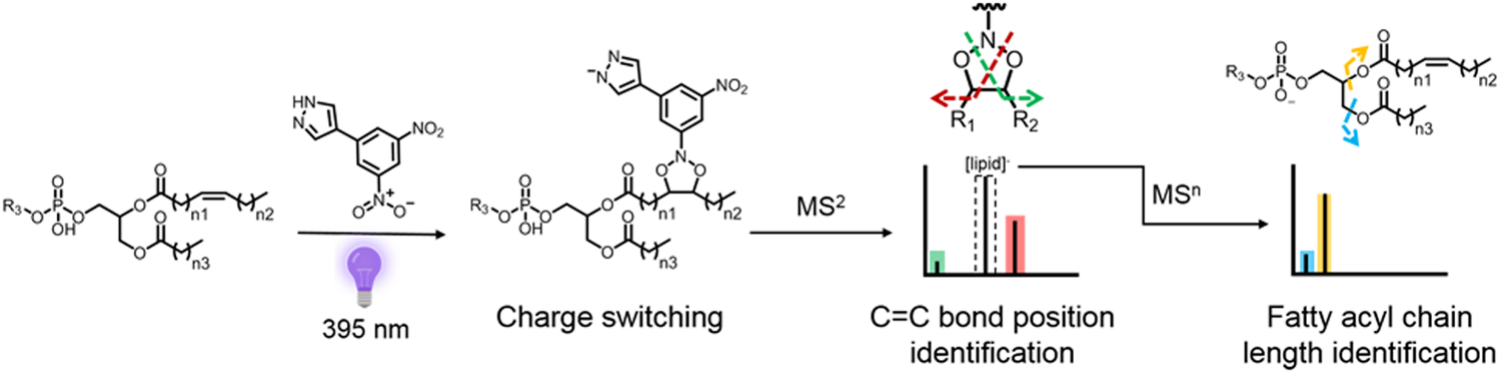

Glycerophospholipids (GPLs) are structurally diverse biomolecules that play crucial roles in cellular membranes, signaling, and metabolism. Electrospray ionization-tandem mass spectrometry (ESI-MS/MS) has been widely used for GPL identification due to its high sensitivity and specificity. However, this method often falls short in distinguishing isomeric lipids, such as those differing in the positions of carbon–carbon double bonds. Additionally, the ion types naturally generated during ESI are not always optimal for lipid detection and identification. In this work, we introduce a novel bifunctional tag, nitrophenyl pyrazole (DNPZ), which reacts with double bonds in lipids to form N-doped ozonides. In-situ tandem MS analysis of these modified lipids enables simultaneous identification of double-bond positional isomers and charge switching, facilitating the acquisition of comprehensive structural information. Our findings demonstrate that this approach significantly improves ionization efficiency of GPLs in negative ion mode and provides detailed insights into fatty acyl chain compositions and double-bond positions in GPLs. We have demonstrated that this method allows for the characterization of various lipid classes, lipids with multiple double bonds as well as polar lipid extracts from complex biological samples without the need for authentic lipid reference standards.

## Introduction

Glycerophospholipids (GPLs) are major components of eukaryotic cells, playing crucial roles in biological functions such as cellular structure, cell adhesion and migration, cellular signaling, and metabolism.^1,2^ Alterations in the composition of GPLs have been associated with a wide range of chronic diseases, including obesity,^3^ heart disease,^4,5^ neurological diseases,^6−8^ and kidney disease.^9^

Understanding the correlation between diseases and lipid profiles requires detailed structural characterization of lipids. GPLs generally consist of a glycerol backbone, a phosphate headgroup, and two fatty acyl chains. The classification of phospholipid classes is based on the functional groups conjugated to the phosphate headgroup at the *sn*-3 position of the glycerol backbone. Examples of such functional groups include choline, ethanolamine, serine, inositol, and glycerol. The two fatty acyl chains are esterified at the *sn*-1 and *sn*-2 positions on the glycerol backbone and can vary in their carbon chain length, as well as the number and positions of carbon–carbon double (C=C) bonds. These variations give rise to distinct molecular species within each lipid class and can result in different GPL isomers.^10^

Shotgun lipid analysis using electrospray ionization-tandem mass spectrometry (ESI-MS/MS) has emerged as a powerful technique for identifying GPL isomers.^11−15^ Collision-induced dissociation (CID) in commonly used MS/MS provides information on the phosphate headgroup and fatty acyl chain length, revealing structural details of GPLs. However, challenges arise in positive ion mode where the dominant polar headgroup loss ion shows a highly intense signal in the mass spectrum, whereas the minor fatty acyl chain is barely detected. Conversely, negative ion mode provides abundant fatty acyl fragment ions, but some polar GPLs, such as phosphatidylcholines (PCs), exhibit low ionization efficiency in negative ion mode, making detailed acyl chain information difficult to obtain.^16^ Various gas-phase charge inversion ion/ion reactions^17−21^ and solution-phase charge switching^22−25^ have been developed to achieve positive-to-negative charge conversion, thereby enhancing ionization efficiency in negative ion mode.^19^ For example, in gas-phase charge inversion studies, GPLs are sprayed in positive ion mode and subjected to gas-phase ion/ion reactions with deprotonated reagents to form negatively charged complex anions. Subsequent CID in MS/MS yields detailed information on fatty acyl chains, including the number of carbons and the degree of unsaturation.^17−21^ On the other hand, in solution-based reactions, bicarbonate, acetate, formate, or chloride anions are often added to lipid solutions, directly forming an adduct anion in negative ion mode. However, these methods rarely determine the position of C=C bonds in individual fatty acyl chains.

To accurately determine the positions of C=C bonds in lipids, chemical derivatization before MS analysis can be highly effective. This approach involves introducing specific chemical groups to lipid molecules, producing unique fragmentation patterns during CID in MS/MS. Advantages of this technique include minimal instrumentation requirements and the accessibility of various derivatizing agents. Derivatization typically targets C=C bonds within the lipid structure, creating bonds with lower energy that break easily during CID, revealing structural details. Available derivatization reactions include Paternò–Büchi (P–B) reaction,^11,26^ epoxidation,^27−33^ ozonolysis,^34−36^ photooxidation,^37^ aziridination,^38,39^ cross-metathesis,^40^ methoxylation,^41^ methylthiolation,^42^ and Diels–Alder reaction.^43^ These reactions selectively modify C=C bonds, allowing for precise localization of C=C bonds through the observed fragmentation patterns in MS spectra. Franklin et al. combined PB reactions for lipid derivatization with ion/ion reactions for charge inversion to achieve C=C bond identification and charge switching using two steps of reactions.^44^

**Figure 1:**
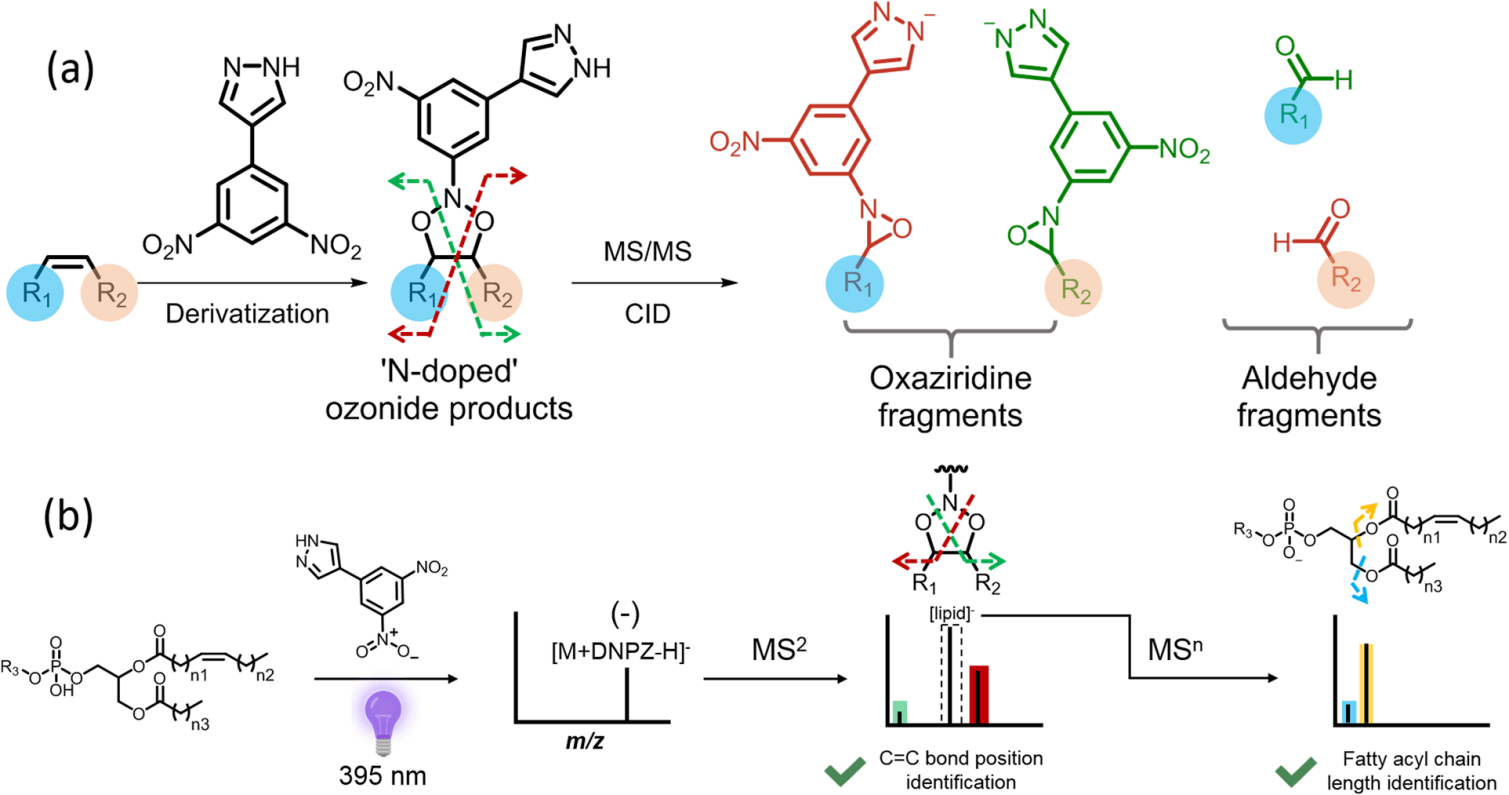
Lipid radical [3 + 2] cycloaddition using DNPZ tag to form N-doped ozonides coupled with tandem mass analysis in negative ion mode, enabling lipid characterization at the C=C bond positional isomer level. (a) Fragmentation of N-doped ozonide products to produce oxaziridine and aldehyde fragments as C=C bond positional diagnostic ions; (b) workflow of lipid DNPZ tagging for charge switching and structure characterization.

In this work, we develop a novel bifunctional tag, dinitrophenyl pyrazole (DNPZ), which can serve as both an effective charge-switching reagent and a photochemical reactant for C=C double bonds through a nitroarene-promoted method.^45,46^ This approach introduces a new and efficient reaction specifically designed for determining the position of C=C bonds in lipids, providing a significant advancement in lipid structural analysis (Figure 1). The tag enables both charge switching and C=C positional identification within a single experiment. The DNPZ tag, synthesized in-house, features two critical functional groups: (i) the pyrazole group, which is readily deprotonated to form a negative ion, and (ii) the nitroarene functional group, which undergoes radical [3 + 2] cycloaddition with alkenes. Our findings demonstrate that the tag enhances the ionization of GPLs and effectively converts positively charged lipids to negatively charged ones, yielding informative structural information on the acyl chains. Fragmentation of the *N*-doped ozonide derivatives in CID produces significant characteristic fragments that reveal C=C bond positions within GPLs. We have applied this method to characterize various lipids from complex biological sample extracts without the use of authentic lipid standards.

## Experimental Section

### Materials and Reagents

All reagents and solvents were purchased from commercial sources and without further purification. Acetonitrile (ACN), water (H_2_O), Dichloromethane (CH_2_Cl_2_), methanol (MeOH), ethyl acetate (EtOAc) and oleic acid were purchased from Thermo Fisher Scientific (Waltham, MA) and Sigma-Aldrich (St Louis, MO). All lipid standards and the soybean polar lipid extract were purchased from Avanti Polar Lipids, Inc. (Alabaster, AL).

### Nomenclature

The lipid nomenclature used here follows the guidelines established by Liebisch et al.^47^ The positions of C=C bonds are indicated in lipid names using numbers in brackets according to the Δ-nomenclature system. Additionally, the sn positions of fatty acyl chains are represented by the symbol “/” for known positions and “_” for unknown positions. For example, PC 16:0_18:1(Δ9) indicates that the *sn* position is not determined and that there is one C=C bond between carbons 9 and 10 from the carbonyl group.

### Synthesis of Dinitrophenyl Pyrazole (DNPZ)

(1H-Pyrazol-4-yl)boronic acid (0.07 g, 0.6 mmol), tricyclohexylphosphine (0.01 g, 0.05 mmol) and Pd_2_(dba)_3_ (0.02 g, 0.02 mmol) were dissolved in dioxane (2 mL) under a nitrogen atmosphere. 1-Bromo-3,5-dinitrobenzene (0.1 mg, 0.4 mmol) was added followed by K_3_PO_4_ (0.17 g, 0.8 mmol) and water (0.5 mL). The reaction was stirred at room temperature for 30 min and then at 90 °C for 20 h, cooled to room temperature and filtered through a glass fiber paper. The solid was washed with ethyl acetate (10 mL) and water (5 mL). The filtrate was extracted with ethyl acetate (5 mL) and the organic phases were washed with brine (5 mL), dried over sodium sulfate, filtered, and concentrated under reduced pressure. The residue was purified by column chromatography on silica gel with hexane/EtOAc (7/3 to 1/1) to give 4-(3,5-dinitrophenyl)-1H-pyrazole (0.03 g, 35%) as off white solid. ^1^H NMR (400 MHz, DMSO-*d*_6_): Δ 13.29 (brs, 1H), 8.84 (d, *J* = 2.0 Hz, 2H), 8.71 (d, *J* = 1.0 Hz, 1H), 8.61 (t, *J* = 2.0 Hz, 1H), 8.33 (d, *J* = 1.3 Hz, 1H) ppm^48,49^ (Supporting Information Section S10 and Figure S38).

### Derivatization of Lipid Standards by Nitroarene

Lipid (2 mM in degased CH_2_Cl_2_, 1 equiv) and DNPZ (3 mM in degased EtOAc, 1.5 equiv) were added to an oven-dried and N_2_-purged vial, followed by the addition of anhydrous and degased CH_2_Cl_2_/EtOAc in a 1:1 ratio to achieve a lipid concentration of 100 μM. The reaction vial was then placed in a photoreactor M2 (Acceled) and stirred under 395 nm irradiation for 1 h, and the light intensity was set to 50% during the reaction. After the reaction, the solvent was removed by rotary evaporation, and the residue was resuspended in ACN to achieve a concentration of 100 μM. The resulting solution was analyzed by nanoESI-MS.

### Derivatization of Lipid Extracts by Nitroarene

Polar lipid extract from soybean was dissolved in CH_2_Cl_2_/EtOAc = 1:1 to achieve a concentration of 0.5 mM, then DNPZ (5 mM, 10 equiv) was added to the solution in an oven-dried vial. The reaction vial was purged with N_2_ for 15 min. The reaction vial was then placed in a photoreactor M2 (purchased from Acceled) and stirred under 395 nm irradiation for 1 h. The light intensity was set to 50% during the reaction. After the reaction, the solvent was removed by rotary evaporation, and the residue was resuspended in ACN to achieve a concentration of 100 μM. The resulting solution was analyzed by nanoESI-MS.

### Mass Spectrometric Analysis

Lipid analysis was conducted using an Orbitrap Fusion mass spectrometer from Thermo Fisher Scientific (San Jose, CA). NanoESI emitters were fabricated from borosilicate glass tubing (WPI Inc., Sarasota, FL) with a P-100 micropipette puller (Sutter Instrument Company, Novato, CA) using the following parameters: heat 554, pull 0, velocity 10, time 250, and pressure 500. Samples were ionized in negative ion mode with spray voltages ranging from 2.0 to 2.5 kV. Full MS scans were acquired over the *m*/*z* range of 400–1500 in negative ion mode, with the microscan number set to 1 and the maximum injection time set to 100 ms. The in-source energy was applied in the range of 0–80 V (Supporting Information, Section S3). CID was used to obtain tandem mass spectra with a normalized collision energy of 30–35. For soybean polar lipid extract analysis, the method was set to collect one MS^2^ scan followed by two MS^3^ scans per cycle, repeating this sequence continuously throughout the 20 min run, allowing the selection of up to 80 different precursor ions for MS^2^ analysis during the acquisition.

## Results and Discussion

### Design of the Bifunctional DNPZ Tag Enabling Simultaneous Charge Switching and C=C Bond Position Determination

**Figure 2:**
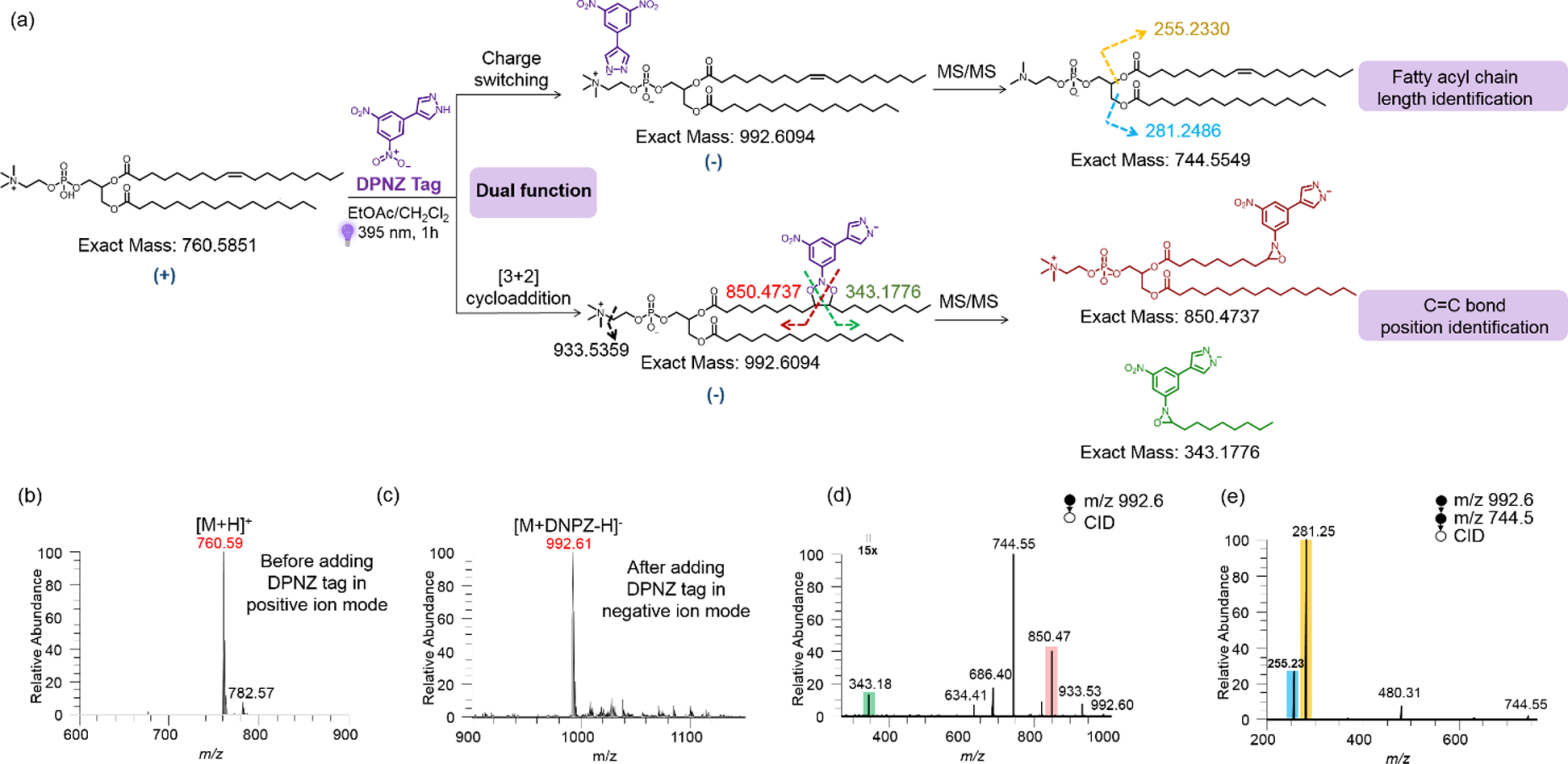
(a) Schematic representation of the dual function of DNPZ tag in analyzing PC 16:0_18:1 (Δ9); full mass spectrum of PC 16:0_18:1 (Δ9) (b) before derivatization in positive mode and (c) after derivatization in negative mode; (d) MS^2^ spectrum of derivatized PC 16:0_18:1 (Δ9)at *m*/*z* 992.6, showing C=C bond position diagnostic ions at *m*/*z* 343.18 and 850.47 in negative ion mode (labeled in green and red); (e) further fragmentation of [PC-CH_3_]^−^ at *m*/*z* 744.5 in MS^3^ spectrum, showing the ions at *m*/*z* 255.23 (C16:0) and 281.25 (C18:1) for fatty acyl chain lengths determine.

The novel bifunctional dinitrophenyl pyrazole (DNPZ) tag incorporates two important functional groups that simultaneously achieve charge switching and C=C bond position identification. First, the pyrazole group in the tag, which acts as the ionization group, can lose a proton to form a negative charge. When the tag is conjugated to a lipid molecule, it converts the entire molecule into a negatively charged species, enabling charge switching (Figures 1b and 2a). The other important functional group in the DNPZ tag is dinitrobenzene. Recent studies have shown that photoexcited nitroarenes can serve as highly efficient substitutes for ozone in addition reactions of C=C bonds of alkenes.^45,46^ This method has been used for anaerobic cleavage of alkenes into carbonyl compounds, with nitroarenes as oxygen transfer reagents. In this study, we take advantage of the five-membered ‘N-doped’ ozonide formed under UV light (395 nm) through the reaction of dinitrobenzene and C=C bonds, prior to alkene cleavage into carbonyl compounds, and have optimized reaction conditions to achieve a maximum yield of 57% (Supporting Information Section S1 and Table S1).

By leveraging the N-doped ozonide product, we not only enhanced the ionization efficiency of GPLs in negative ion mode to obtain intense fatty acyl chain signals, but also enabled the characterization of C=C bond positions through tandem MS cleavage (Figure 1a). In this method, GPL and DNPZ were mixed in CH_2_Cl_2_/EtOAc (1:1) under nitrogen purging to achieve a final concentration of 100 μM. The [3 + 2] cycloaddition reaction was initiated by 395 nm UV irradiation for 1 h, followed by nanoESI analysis in negative ion mode. The resulting adducts were fragmented via CID, producing oxaziridine and aldehyde fragments that pinpoint the C=C bond positions in the GPLs (Figure 1a). The DNPZ tag can be deprotonated to form a negative ion during MS analysis, facilitating positive-to-negative charge switching. Subsequent fragmentation of the product ion enables the determination of both the C=C bond positions and the acyl chain lengths in GPL isomers. This tandem MS analysis provides a comprehensive approach for detailed structural characterization of GPLs (Figure 1b).

PC 16:0_18:1 (Δ9) was utilized as a standard lipid to demonstrate the capability of DNPZ in achieving charge switching and C=C positional identification (Figure 2a). After reacting with DNPZ, the derivatized lipid was detected at *m*/*z* 992.61 by nanoESI-MS (Figure 2b), which contains lipid ion cluster and [3 + 2] cycloaddition product. To assess the charge-switching efficiency of our tag in converting the derivatized lipid to a negative charge, we calculated the operational efficiency,^50^ which was approximately 50% (Supporting Information Section S2). Fragmentation of ions at *m*/*z* 992.61 in CID generated a pair of diagnostic oxaziridine ions at *m*/*z* 343.18 and *m*/*z* 850.47, indicating the C=C bond position at Δ9. The aldehyde fragment ion at *m*/*z* 634.41 was also found in the MS^2^ spectrum, further confirming the C=C bond position at Δ9. Additionally, the neural loss of the trimethylamine from the headgroup was observed at *m*/*z* 933.53, aiding in characterizing the GPL subclass as PC (Figure 2d).

Interestingly, the [PC–CH_3_]^−^ ion at *m*/*z* 744.55 was observed in the MS^2^ spectrum. This indicates ions at *m*/*z* 992.61 contain both cycloaddition product and lipid ion cluster with DNPZ tag (Figure 2a,d and Supporting Information Section S4). The lipid ion cluster facilitates the fatty acyl chain length determination as further fragmentation of it via CID generated abundant fatty acyl anions at *m*/*z* 255.23 and *m*/*z* 281.25, indicating the presence of C16:0 and C18:1 acyl chains (Figure 2e).

The limit of detection (LOD) of the DNPZ tagging method was evaluated using PC 16:0_18:1(Δ9) under two reaction conditions. At room temperature, diagnostic ions for the C=C bond position were clearly observed in the MS^2^ spectra at a concentration of 1 μM, with a signal-to-noise ratio above 3 (Supporting Information Section S5 and Figure S7). This condition was selected for its practicality and ease of implementation in routine workflows. In order to improve the efficiency of the radical [3 + 2] cycloaddition reaction, we also conducted the reaction at −10 °C, which resulted in an improved LOD of 150 nM (Supporting Information Section S5 and Figure S9). These results demonstrate that DNPZ tag effectively converts positively charged lipids into negatively charged lipid ion clusters and enables the formation of cycloaddition products at C=C bonds. In-situ tandem MS analysis of these products allows for comprehensive characterization of the headgroup, acyl chain lengths, and C=C bond positions in GPLs.

### Feasibility of DNPZ Tag Reactions with Lipids Featuring Diverse C=C Bond Positions, Polyunsaturation Levels, and Head Groups

To assess the broad applicability of our method, we extended the use of the DNPZ tag to identify lipids with varying C=C bond positions, degrees of polyunsaturation, and a range of head groups.

**Figure 3:**
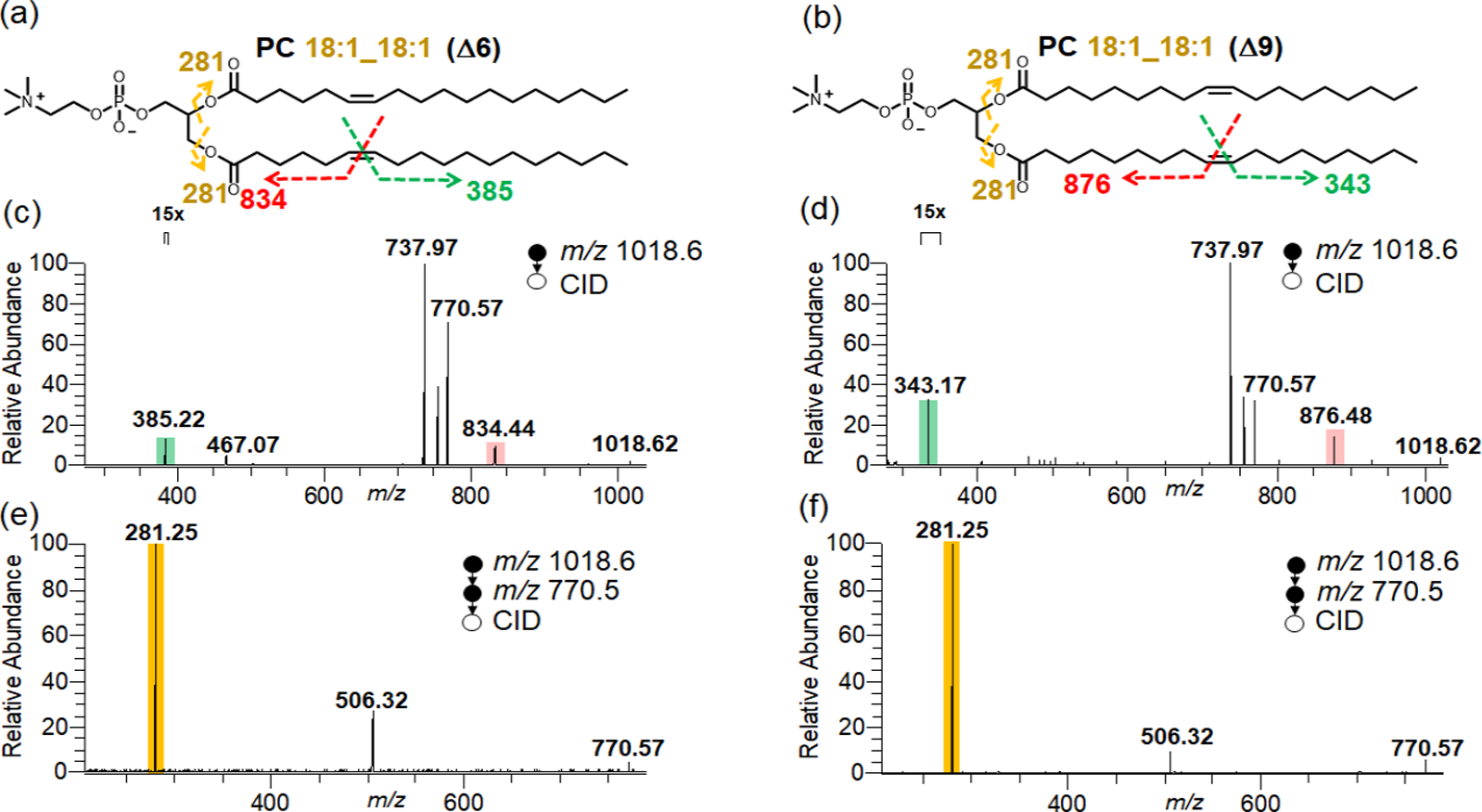
Diagnostic fragmentation indicated in the structure of (a) PC 18:1_18:1 (Δ6) and (b) PC 18:1_18:1 (Δ9); MS^2^ spectrum of (c) derivatized PC 18:1_18:1 (Δ6) at *m*/*z* 1018.6, showing C=C bond position diagnostic ions at *m*/*z* 385.22 and 834.44 and (d) derivatized PC 18:1_18:1 (Δ9) at *m*/*z* 1018.6, showing C=C bond position diagnostic ions at *m*/*z* 343.17 and 876.48; further fragmentation of [M-CH_3_]^−^ at *m*/*z* 770.5 in MS^3^ spectrum of (e) PC 18:1_18:1 (Δ6) and (f) PC 18:1_18:1 (Δ9), producing the diagnostic ions at *m*/*z* 281.25, confirming fatty acyl chain as C18:1.

**Figure 4:**
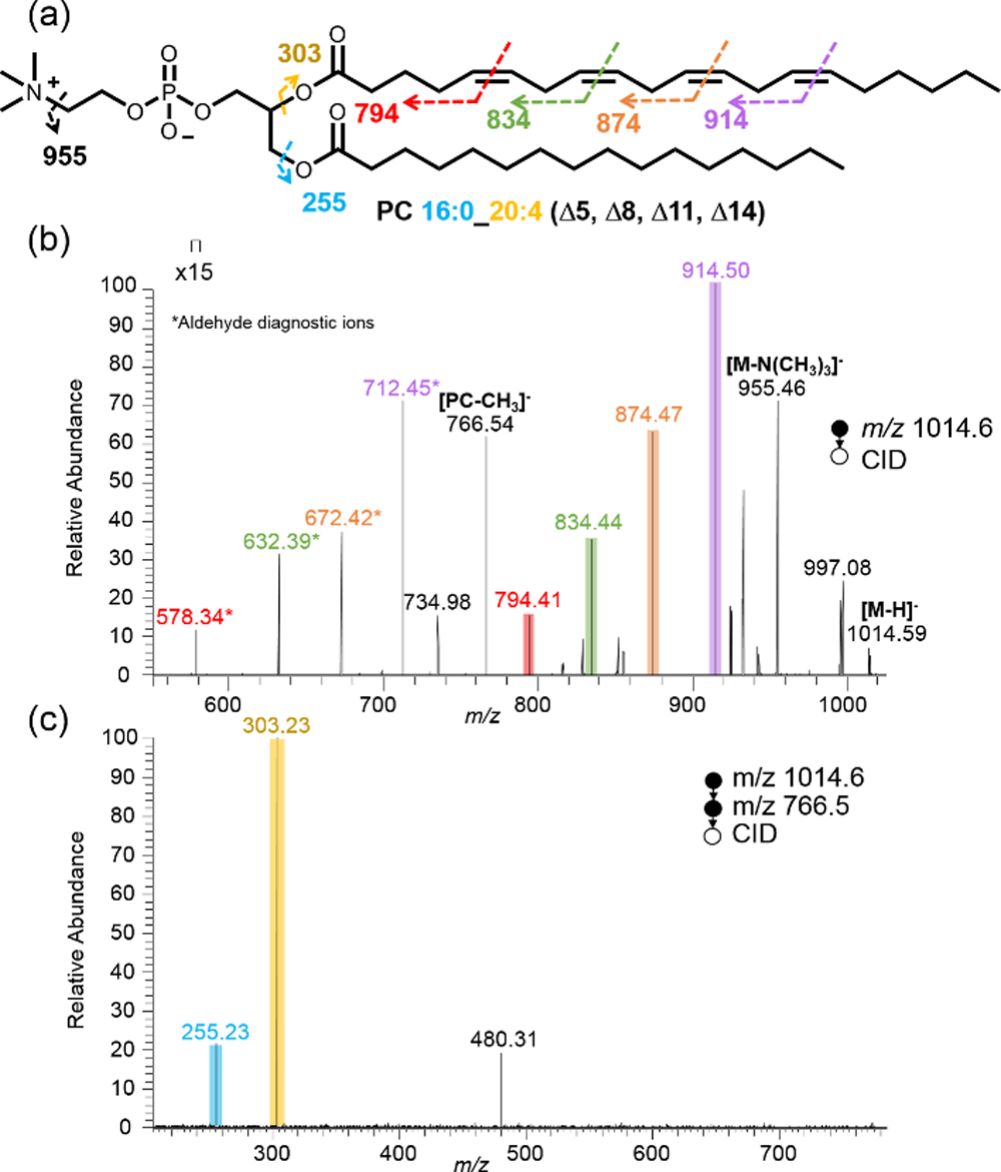
(a) Diagnostic fragmentation indicated in the structure of PC 16:0_20:4 (Δ5, Δ8, Δ11, Δ14) (b) CID spectrum of derivatized PC 16:0_20:4 (Δ5, Δ8, Δ11, Δ14), showing the diagnostic ions at *m*/*z* 794.41(Δ5), 834.44 (Δ8), 874.47 (Δ11), 914.50 (Δ14) (c) Further fragmentation of [PC-CH_3_]^−^ at *m*/*z* 766.5 in MS^3^ spectrum producing the acyl chain fragment ions at *m*/*z* 255.23 (C16:0) and 303.23 (C20:4).

We first analyzed a pair of C=C bond positional isomers, PC 18:1(Δ6) _18:1 (Δ6) and 18:1 (Δ9)_18:1 (Δ9) using the DNPZ tag (Figure 3a,b). Clearly, two different pairs of diagnostic oxaziridine ions were observed at *m*/*z* 385.22 and 834.44, and *m*/*z* 343.17 and 876.48 by nanoESI-MS/MS, corresponding to the C=C bond positions at Δ6 and Δ9, respectively (Figure 3c,d). Further fragmentation of [PC–CH_3_]^−^ion at *m*/*z* 770.57 via CID generated abundant fatty acyl anions at *m*/*z* 281.25, indicating the presence of C18:1 acyl chains (Figure 3e,f). These results demonstrate that the DNPZ tag can successfully differentiate the positions of C=C bonds in lipid isomers. Quantitative analysis was performed using a series of mixtures of PC 18:1(Δ6)_18:1(Δ6) and PC 18:1(Δ9)_18:1(Δ9). The calibration curve was generated by plotting the intensity ratios of diagnostic ions against the concentration ratios of the lipid isomers. The intensity ratio was calculated as (I_834_ + I_385_)/(I_876_ + I_343_), where I represents the ion intensities. The total lipid concentration was maintained at 100 μM, while the concentration ratios of the isomers were varied from 1:9 to 9:1. The resulting ion intensity ratios of the diagnostic ion pairs showed a strong linear correlation with the isomer concentration ratios (C_6_/C_9_), yielding an *R*^2^ value of 0.9979 (Figure S10). Next, we challenged the DNPZ tagging with polyunsaturated GPLs containing multiple C=C bonds, using PC 16:0_20:4 (Δ5, Δ8, Δ11, Δ14) as a model (Figure 4a). A derivatized lipid solution at 100 μM in ACN was analyzed using negative ion mode nanoESI-MS/MS. The derivatized lipid was observed at *m*/*z* 1014.59. CID of this product ion generated a set of diagnostic ions in the MS^2^ spectrum, including *m*/*z* 794.41, 834.44, 874.47, and 914.50 (Figure 4b). Notably, these diagnostic ions exhibited high intensity, attributed to the formation of N-doped species. Additionally, a set of aldehyde diagnostic ions, *m*/*z* 578.34, 632.39, 672.42, and 712.45, was also detected in the spectrum (Supporting Information Section S7). All these abundant fragmentation ions confidently identified the C=C bond positions at Δ5, Δ8, Δ11, and Δ14. Interestingly, the spectrum displayed varying intensities for different positional diagnostic ions, which can be attributed to the different reactivity of alkenes. Terminal alkenes (e.g., Δ14) exhibited greater reactivity and thus higher intensity compared to internal alkenes (e.g., Δ5). Further CID of the ions at *m*/*z* 766.5 [lipid-CH_3_]^−^produced diagnostic ions at *m*/*z* 255.23 and 303.23, confirming the fatty acyl chains as C16:0 and C20:4.

**Figure 5:**
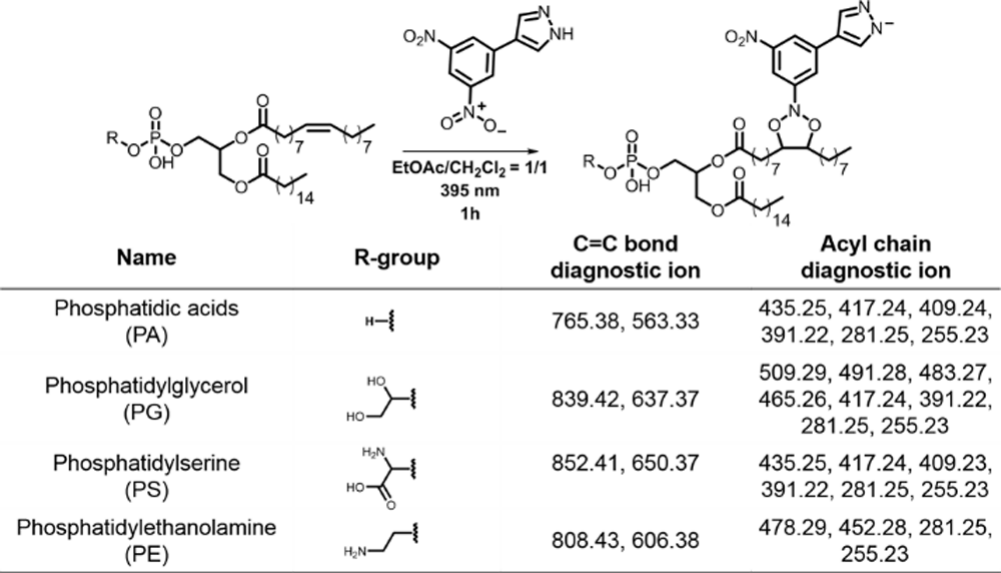
Diagnostic ions observed upon CID for determination of C=C bond positions and acyl chain lengths in GPLs, PA, PG, PS PE.

We then examined the applicability of DNPZ tagging across various GPL subclasses, including PA, PG, PS and PE. The MS^2^ spectra of these GPLs produced diagnostic ions that indicated the positions of C=C bonds, while subsequent MS^n^ spectra provided fragments of fatty acyl chains (Figure 5 and Supporting Information Section S8). For instance, PA 16:0_18:1 (Δ9) produced diagnostic ions at *m*/*z* 765.38 and 563.33 upon CID fragmentation, confirming the C=C bond at Δ9 in the fatty acyl chain. Further MS^3^ analysis in CID yielded fatty acyl chain ions at *m*/*z* 255.23, 281.25, 391.22, 409.24, 417.24 and 435.25, indicating the fatty acyl chains C16:0 and 18:1. In addition to PG, PS and PE also exhibited diagnostic ions in the MS^2^ spectrum. MS^3^ fragmentation of PG produced a fragment ion at *m*/*z* 673.48 (−74 Da, [PG-glycerol]^−^), indicating the neutral loss of the glycerol group. The MS^3^ spectrum of PS showed a neutral loss of the serine group, yielding an ion at *m*/*z* 673.48 (−87 Da, [PS-serine]^−^). Further isolation and fragmentation of the *m*/*z* 673.48 ion showed fatty acyl chain ions in the MS^4^ spectrum, specifically at *m*/*z* 255.23 and 281.25. Although PE does not exhibit any neutral loss in either the MS^2^ or MS^3^ spectrum, the presence of fatty acyl chain ions in the MS^3^ spectrum at *m*/*z* 255.23, 281.25, 452.28, and 478.29 can be used to deduce headgroup information. Specifically, the ions at *m*/*z* 452.28 and 478.29 correspond to fragment ions formed by the loss of one fatty acyl chain, retaining the PE headgroup. These fragment ions, combined with the characteristic fatty acyl ions at *m*/*z* 255.23 and 281.25, allow for the identification of the PE headgroup.

### Application of DNPZ Tag for Characterizing Lipids in Soybean Polar Lipid Extracts

Lastly, we applied our method to the analysis of lipid extracts from soybean, obtained from Avanti.^30^ The lipid extract was dissolved in CH_2_Cl_2_/EtOAc (1:1) to achieve a concentration of 0.5 μg/μL (500 μM). The lipid solution was mixed with DNPZ (5 mM) and reacted for 1 h under UV light before the mixture was subjected to nanoESI-MS using data-dependent acquisition (DDA). After derivatization, lipids with an additional 233 Da were observed in the *m*/*z* range of 900–1100 in negative ion mode. Fragmentation of the derivatized products provided diagnostic ions or characteristic fragment ions for C=C bond positional and headgroup determination. Additionally, further CID of the lipid ions produced structural information on the fatty acyl chains. Characterization of PC 36:4 was illustrated as an example. The derivatized lipid was observed at *m*/*z* 1014.60, and CID produced ions at *m*/*z* 874.47 and 914.50, indicating the locations of C=C bonds at Δ9 and Δ12 in the fatty acyl chain. Other fragment ions at *m*/*z* 955.50 (−59 Da) indicated the loss of the choline group. Further CID of the lipid ion produced fragmentation ions at *m*/*z* 279.23 and 518.29, resulting from the fatty acyl chains C18:2 and C18:2. Therefore, PC 36:4 was determined to be PC 18:2(Δ9, Δ12)_18:2(Δ9, Δ12). In total, using our method, we successfully identified 31 lipids from the soybean polar lipid extract. The positions of multiple C=C bonds were assigned within a single acyl chain or distributed between two different chains (Table 1 and Supporting Information Section S9).

**Table 1:** Identified Lipids with Assigned C=C Bond Positions in Soybean Polar Lipid Extracts

	forms	exact mass	derivatized	C=C positional diagnostic ion	fatty acyl chain diagnostic ion	identified lipids
PC 34:2	[M – H]^−^	756.5549	990.5938	890.49, 850.46 and 648.37	279.23 and 255.23	PC 16:0_18:2(Δ9, Δ12)
PC 34:3	[M – H]^−^	754.5392	988.5781	930.50, 890.53 and 850.50	277.22 and 255.23	PC 16:0_18:3(Δ9, Δ12, Δ15)
PC 36:4	[M – H]^−^	780.5549	1014.5938	914.50 and 874.47	279.23	PC 18:2(Δ9, Δ12)_18:2(Δ9, Δ12)
PC 36:5	[M – H]^−^	778.5392	1012.5781	954.53, 914.47, 912.49, 874.47 and 872.46	279.23 and 277.22	PC 18:3(Δ9, Δ12, Δ15)_18:2 (Δ9, Δ12)
PE 34:2	[M – H]^−^	714.5079	948.5468	848.46, 808.42, 646.41 and 606.38	279.23 and 255.23	PE 16:0_18:2(Δ9, Δ12)
PE 36:4	[M – H]^−^	738.5079	972.5468	872.46, 832.42, 630.38 and	279.23	PE 18:2(Δ9, Δ12)_18:2(Δ9, Δ12)
PE 36:5	[M – H]^−^	736.4923	970.5312	912.49, 872.46, 870.44, 832.42, 830.41 and 628.36	279.23 and 277.22	PE 18:3(Δ9, Δ12, Δ15)_18:2(Δ9, Δ12)
PE 37:3	[M – H]^−^	754.5392	988.5781	888.45, 884.42, 686.40, 646.37 and 630.38	295.23 and 279.23	PE 18:2(Δ9, Δ12)_19:1(Δ9)
Lysp PI 16:0	[M – H]^−^	571.2889	-	-	391.22 and 255.23	Lyso PI 16:0
PI 34:2	[M – H]^−^	833.5186	1067.5575	967.47, 927.43, 765.42 and 725.44	279.23 and 255.23	PI 16:0_18:2(Δ9, Δ12)
PI 34:3	[M – H]^−^	831.5029	1065.5418	1007.50, 967.47, 927.43, 805.45, 765.42 and 725.39	277.22 and 255.23	PI 18:3(Δ9, Δ12, Δ15)_16:0
PI 36:4	[M – H]^−^	857.5186	1091.5575	991.47, 951.44, 789.42 and 749.52	279.23	PI 18:2(Δ9, Δ12)_18:2(Δ9, Δ12)
PA 34:2	[M – H]^−^	671.4657	905.5046	805.41, 765.38, 563.33	279.23 and 255.23	PA 16:0_18:2(Δ9, Δ12)
PA 34:3	[M – H]^−^	669.4501	903.4890	845.45, 831.43, 805.41, 803.40, 789.38, 765.38 and 763.37	279.23, 277.22, 255.23 and 253.22	PA 18:3(Δ9, Δ12, Δ15)_16:0
						PA 18:2(Δ9, Δ12)_16:1(Δ9)
						PA 18:2(Δ9, Δ12)_16:1(Δ12)
						PA 18:2(Δ12, Δ15)_16:1(Δ9)
						PA 18:2(Δ12, Δ15)_16:1(Δ12)
PA 36:4	[M – H]^−^	695.4657	929.5044	829.41, 789.38 and 587.33	279.23	PA 18:2(Δ9, Δ12)_18:2(Δ9, Δ12)
PA 36:5	[M – H]^−^	693.4501	927.4890	869.45, 829.41, 827.40, 789.38 and 787.37	279.23 and 277.22	PA 18:3(Δ9, Δ12, Δ15)_18:2(Δ9, Δ12)
PA 37:3	[M – H]^−^	711.4970	945.5459	845.41, 805.38 and 789.38	295.23 and 279.23	PA 18:2(Δ9, Δ12)_19:1 (Δ9)
PA 37:4	[M – H]^−^	709.4814	943.5203	885.44, 845.41, 843.39, 829.31, 805.38, 803.36, 789.38 and 787.37	295.23, 293.21, 279.23 and 277.22	PA 18:2 (Δ9, Δ12)_19:2 (Δ9, Δ12)
						PA 18:3 (Δ9, Δ12, Δ15)_19:1 (Δ9)
						PA 18:3 (Δ9, Δ12, Δ15)_19:1 (Δ12)
PG 34:1	[M – H]^−^	748.5254	981.5571	881.46 and 839.42	281.25 and 255.23	PG 18:1(Δ9)_16:0
						PG 18:1(Δ12)_16:0
PG 34:2	[M – H]^−^	745.5025	979.5414	879.43, 839.42 and 637.37	279.23 and 255.23	PG 16:0_18:2(Δ9, Δ12)
						PG 16:0_18:2(Δ9, Δ15)
PG 35:1	[M – H]^−^	761.5338	995.5727	895.45 and 839.42	295.23 and 255.23	PG 19:1(Δ9)_16:0
						PG 19:1(Δ13)_16:0
PG 36:4	[M – H]^−^	769.5020	1003.5414	903.45, 863.43 and 661.36	279.23	PG 18:2(Δ9, Δ12)_18:2(Δ9, Δ12)

## Conclusions

In this study, we have developed a novel bifunctional DNPZ tag for charge switching and structural characterization of GPLs at the C=C bond positional isomer level. The DNPZ tag can be easily deprotonated to form a negative ion, facilitating positive-to-negative charge switching in MS and overcoming the suppression of acyl chain length ions in positive tandem MS. DNPZ tag also allows forming N-doped lipid ozonide through radical [3 + 2] cycloaddition, which produces diagnostic oxaziridine fragments to determine C=C bond positions. This method has been demonstrated using both lipid standards and lipid extracts from complex biological samples such as soybean polar extract. The DNPZ tag successfully characterized multiple C=C bonds in polyunsaturated lipids and was applicable across various subclasses of GPLs, including PC, PE, PI, PG, PS, and PA. The ability to assign C=C bond positions, identify fatty acyl chain compositions, and determine headgroup types demonstrates the versatility and robustness of this method. A limitation of this method is its relatively high limit of detection, approximately 1 μM at room temperature, but this can be improved to 150 nM at −10 °C, expanding its potential for detecting lower-abundance species under optimized conditions. Despite this, the method provides a significant advantage by enabling simultaneous double bond localization and acyl chain identification in negative ion mode with enhanced diagnostic ion intensity. Overall, the DNPZ tagging method provides a powerful tool for detailed lipid structural analysis, offering significant potential for advancing lipidomics research and contributing to our understanding of lipid-related biological processes and diseases.

## Supporting Information Available

Reaction condition optimization, PC adduct and derivatization with DNPZ, in source energy optimization, calibration curve, and tandem mass data for structural characterization of glycerophospholipids (PDF)
