# Supplementary material for: Bifunctional Tagging through N-Doped Ozonide for Charge Switching and Isomeric Characterization of Glycerophospholipids Using Tandem Mass Spectrometry
Source: Anal Chem. 2025 Apr 17;97(16):8992–9. doi: 10.1021/acs.analchem.5c00443 (PMC12044587; doi:10.1021/acs.analchem.5c00443)
Supplement: Supplementary file 1 — ac5c00443_si_001.pdf [file ac5c00443_si_001.pdf]

Supporting Information

Bifunctional Tagging Through N-doped Ozonide for Charge Switching and Isomeric Characterization of Glycerophospholipids Using Tandem Mass Spectrometry

Chia-Lung Tsai,[a] Xi Chen,[a] Ramidi G Reddy,[a] and Xin Yan[a,*]

[a] Department of Chemistry, Texas A&M University, 580 Ross St., College Station, TX 77843, USA

Table of Content

Section S1. Reaction condition optimization S2

Section S2. The scope of charge-switching methods and transformation efficiency investigation S3

Section S3. In source energy optimization S5

Section S4. The fraction of PC adduct and derivatization with DNPZ S6

Section S5. Limit of detection S8

Section S6. Calibration curve S11

Section S7. Proposed structures of aldehyde diagnostic ions S12

Section S8. Tandem mass spectra for structural characterization of different GPL subclass standards S14

Section S9. Tandem mass spectra for structural characterization of glycerophospholipids in soybean polar lipid extracts S18

Section S10. NMR spectra S40

References S41

Section S1. Reaction condition optimization

The reaction conditions reported in previous literature for achieving the anaerobic cleavage of alkenes into carbonyl compounds involved conducting the reaction in acetonitrile at 23 °C for 12 to 24 hours.[1] To optimize the formation of five-membered 'N-doped' ozonides through the reaction of dinitrophenyl pyrazole (DNPZ) with glycerophospholipids (GPLs), we used fatty acid (FA) 18:1 (Δ9), a key component of GPLs, as a model substrate to refine the reaction conditions and maximize yield. The reaction conditions for unsaturated lipids were optimized by monitoring the conversion ratio of the derivatized product at different reaction times. FA 18:1 (Δ9) (1.0 eq) and DNPZ (1.5 eq) were dissolved in EtOAc (30 mM). The reaction was conducted at room temperature for up to 2 hours, with samples taken at 5, 10, 30, 60, and 120 minutes for analysis. After the reaction, the resulting solution was diluted to 100 μM with ACN and was loaded into nanoESI emitters for MS analysis in negative ion mode. The product ion appeared at *m/z* 515.28 in the spectrum. The conversion ratio was calculated by the relative ion intensity of the product ions divided by the ion intensity of unreacted lipids. The results indicated that a reaction time of 60 minutes yielded the highest conversion rate of 57% (Table S1) compared to other reaction times. Therefore, the 60-minute reaction time was used for subsequent derivatization studies of GPLs.

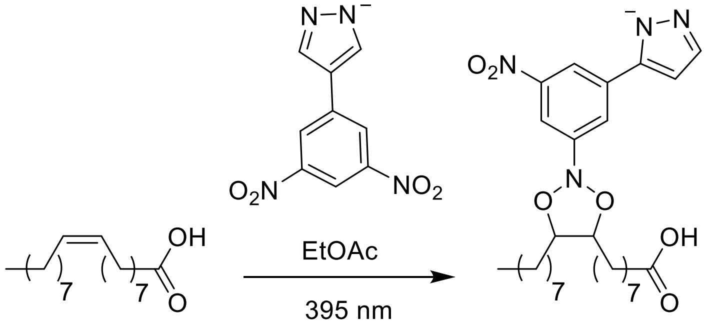

Table S1. The reaction condition and conversion ratio of FA derivatization

FA 18:1	DNPZ	Concentration	Time (min)	Conversion rate
1 eq	1.5 eq	30 mM	0	0%
			5	20%
			10	42%
			30	51%
			60	57%
			120	55%

Section S2. The scope of charge-switching methods and transformation efficiency investigation

Solution-based charge-switching methods typically involve introducing a fixed-charge or easily ionizable group to the analyte prior to MS analysis, and are categorized into two main strategies: (1) noncovalent complexation with metal cations added to the ESI solution (online), and (2) covalent derivatization at specific functional groups using wet-chemical methods (offline). Our DNPZ derivatization approach falls within the second category. While it does not involve gas-phase chemistry, it is consistent with the broader definition of solution-phase charge switching as described in the review.[2] The operational efficiency evaluates the efficiency of the derivatized PC product switching to a negative charge. The equation follows the reported approach.[3]

$$\text{Operational efficiency}=\frac{[\mathrm{PC-CH_3-H}]+\sum([\text{Product related ion}])}{([\mathrm{PC_i+H}]+[\mathrm{PC_i+Na}])-([\mathrm{PC_f+H}]+[\mathrm{PC_f+Na}])}$$

$$=\frac{(5.99\times10^{6})+[(4.85\times10^{5})+(1.06\times10^{6})]}{(1.37\times10^{8}+2.09\times10^{7})-(1.36\times10^{8}+7.85\times10^{6})}\times 100=54\%$$

In this equation, $[\mathrm{PC-CH_3-H}]^-$ represents the intensity of the demethylated PC ion in the full spectrum acquired in negative ion mode, while [Product related ion] refers to the intensity of the derivatized PC product ion in the same mode. $[\mathrm{PC_i}]$ represents the intensity of the PC ion before the reaction in the full spectrum acquired in positive ion mode, and $[\mathrm{PC_f}]$ represents the intensity of the PC ion after the reaction, also in positive ion mode.

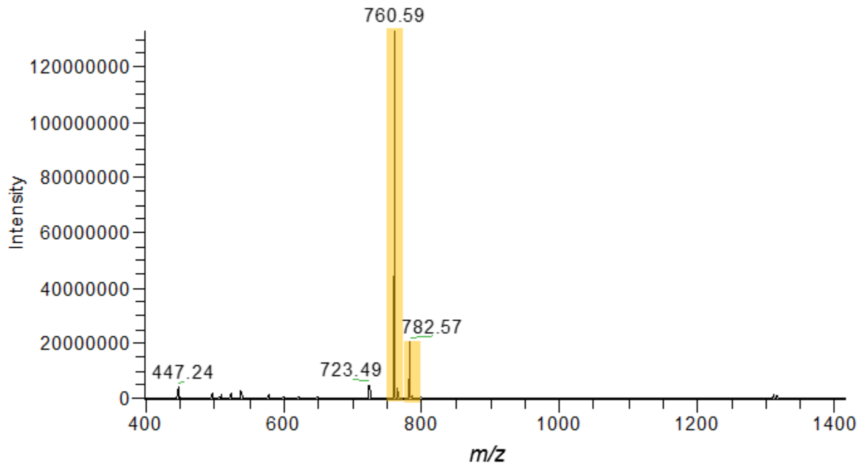

Figure S1. The spectrum of PC 16:0_18:1 (Δ9) before derivatization with the DNPZ tag in positive ion mode. The PC-related ions are at *m/z* 760.59 and 782.57.

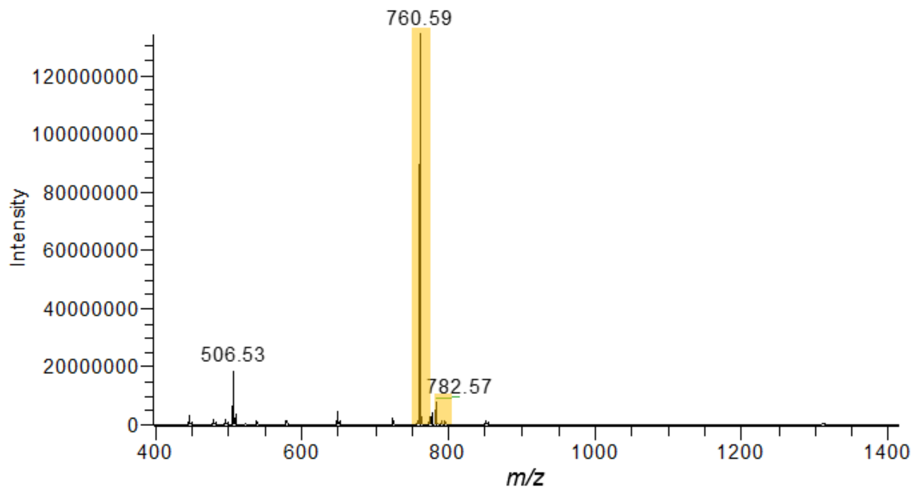

Figure S2. The spectrum of PC 16:0_18:1 (Δ9) after derivatization with the DNPZ tag in positive ion mode. The PC-related ions are at *m/z* 760.59 and 782.57.

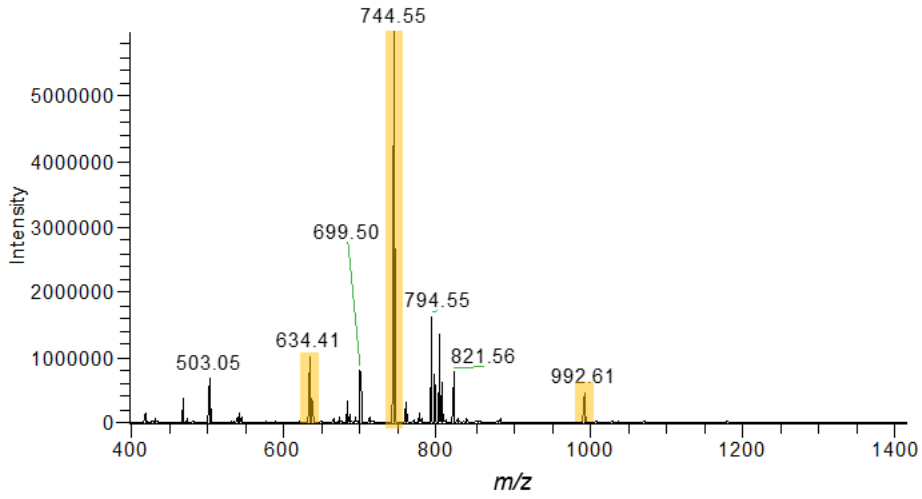

Figure S3. The spectrum of PC 16:0_18:1 (Δ9) after derivatization with the DNPZ tag in negative ion mode. The PC-related ions are at *m/z* 794.55 and 821.56. The PC-derivatization-related ions are at *m/z* 634.41, 744.55 and 992.61.

Section S3. In source energy optimization

The in source energy can remove the PC/DNPZ adduct ion to enhance the intensity of diagnostic ion (DI) in the spectra. The in source energy have been increased from 0V to 80V, the intensity of diagnostic ion at *m/z* 850.47 have also been increased.

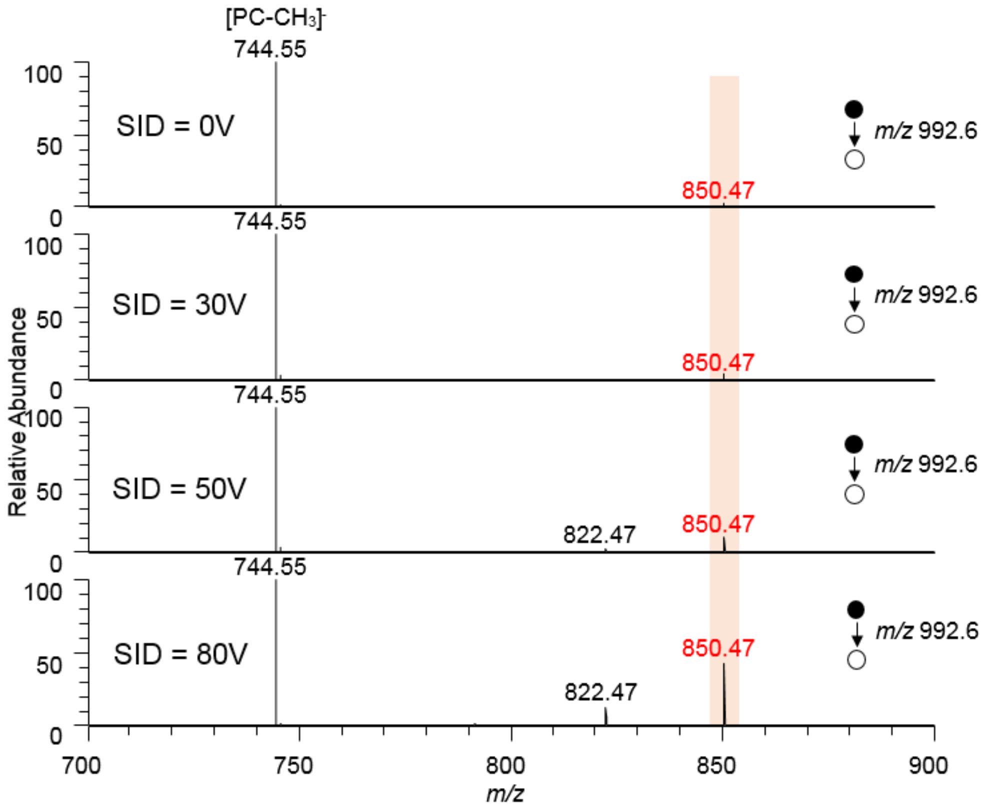

Figure S4. The CID spectra of derivatized PC 16:0_18:1 (Δ9), the intensity of diagnostic ion (DI) at *m/z* 850.47 have been increased when in source energy increased.

Section S4. The fraction of PC adduct and derivatization with DNPZ

The MS^2 spectra before and after the derivatization of PC 18:1 (Δ9)_18:1 (Δ9) are shown in Figure S5 and S6, respectively. In the spectrum before derivatization, the PC formed an adduct with DNPZ, so no diagnostic ions are present. In contrast, after the reaction, diagnostic ions were observed in the spectrum at *m/z* 343.18, 634.41, and 850.47, demonstrating that N-doped ozonide of PC was formed.

The fraction of the non-covalent complex and the [3+2] addition products was determined by comparing the intensity difference of the ions at *m/z* 744.55 before and after the reaction. Prior to derivatization, the ion at *m/z* 992.61 in the spectrum corresponds to the non-covalent complex. After isolation and fragmentation, the MS^2 spectrum shows an ion at *m/z* 744.55 with an intensity of 4.95×10^4, originating from the non-covalent complex. After the derivatization, the intensity of the *m/z* 744.55 ion in the MS^2 spectrum decreased to 2.34×10^4. Therefore, the intensity change of the *m/z* 744.55 ion before and after the reaction can be used to estimate the relative abundance of the two products. The ratio of the [3+2] addition products to the non-covalent complex is 0.9.

$$\text{Conversion ratio} = \frac{I_{744\ before\ rxn} - I_{744\ after\ rxn}}{I_{744\ before\ rxn}} \times 100 = \frac{4.95\times10^4 - 2.34\times10^4}{4.95\times10^4} \times 100 = 52.7\%$$

where the $I_{744\ \text{before rxn}}$ represents the intensity of $[PC\text{-}CH_3]^-$ in the MS^2 spectrum before the reaction, and $I_{744\ \text{after rxn}}$ represents the intensity of $[PC\text{-}CH_3]^-$ in the MS^2 spectrum after the reaction.

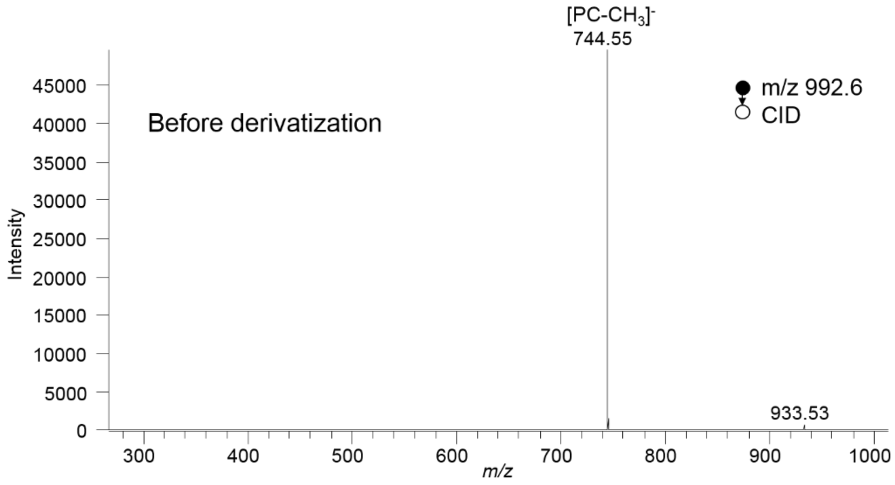

Figure S5. The CID spectrum of lipid before derivatization. PC forms an adduct with DNPZ, so no diagnostic ions are present.

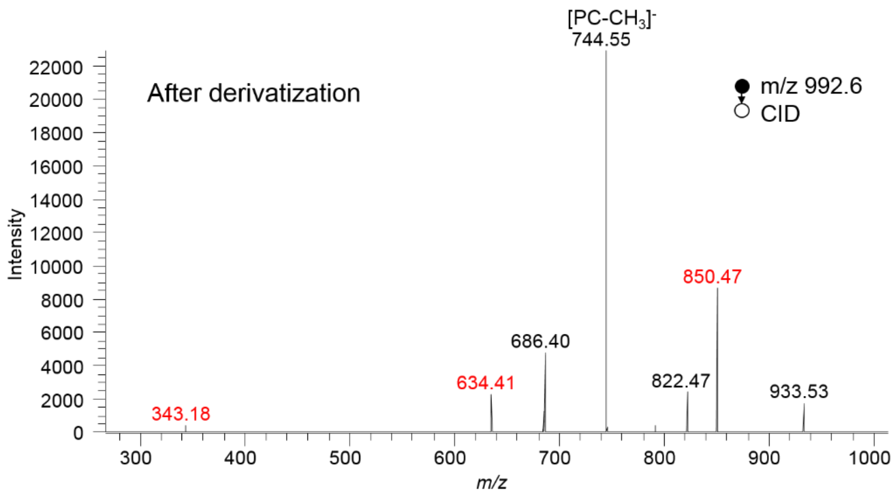

Figure S6. The CID spectrum of lipid after derivatization. The diagnostic ions show in the spectrum at *m/z* 343.18, 634.41, and 850.47

Section S5. Limit of detection

The limit of detection for this method was evaluated using PC 16:0_18:1(Δ9). At a lipid concentration of 1 μM, the diagnostic ions (*m/z* 850.47) were successfully produced and could be observed in tandem mass spectra (Figure S7).

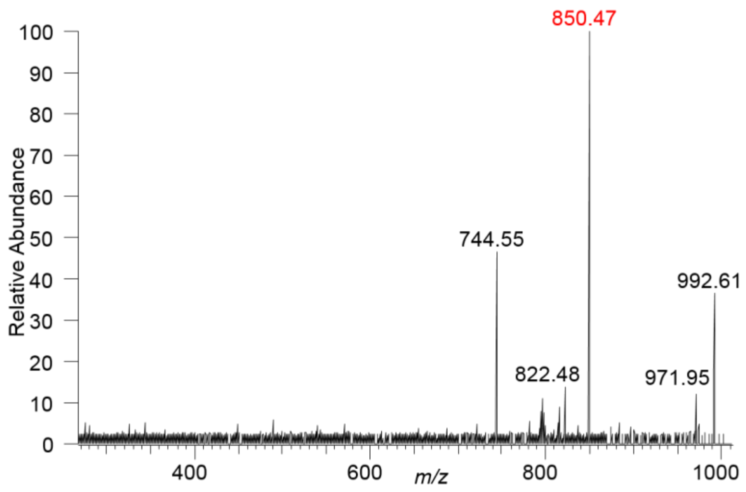

Figure S7. The MS^2 spectrum of derivatized PC 16:0_18:1 (Δ9) (*m/z* 992.6) at 1 μM, producing diagnostic ions *m/z* 850.47 in negative ion mode.

LOD Study: The limit of detection (LOD) of the DNPZ tagging method was evaluated using PC 16:0_18:1(Δ9) under two reaction conditions. The ambient temperature condition, which offers ease of handling and is more compatible with routine workflows, is described in the main text. The relatively high LOD (1 μM) at ambient temperature is attributed to the relatively low efficiency of the radical [3+2] cycloaddition reaction. To demonstrate the potential for improving detection sensitivity, we further optimized the reaction by performing the tagging step at a reduced temperature (−10 °C). PC (1 equiv.) and DNPZ (1.5 equiv.) were added to an oven-dried vial, which was connected to a Schlenk line and subjected to three cycles of vacuum and nitrogen. Anhydrous CH_2Cl_2/EtOAc (1:1, v/v) was then added to the vial. The vial was cooled to −10 °C, and a 390 nm LED lamp was turned on to initiate the photoreaction. The reaction mixture was gently stirred under irradiation and nitrogen protection at −10 °C for 2.5 hours. The mixture was subsequently diluted with acetonitrile and analyzed by nanoESI-MS. The LOD of 150 nM was achieved as evidenced by the clear detection of diagnostic C=C ions in the MS^2 spectra (Figure S9).

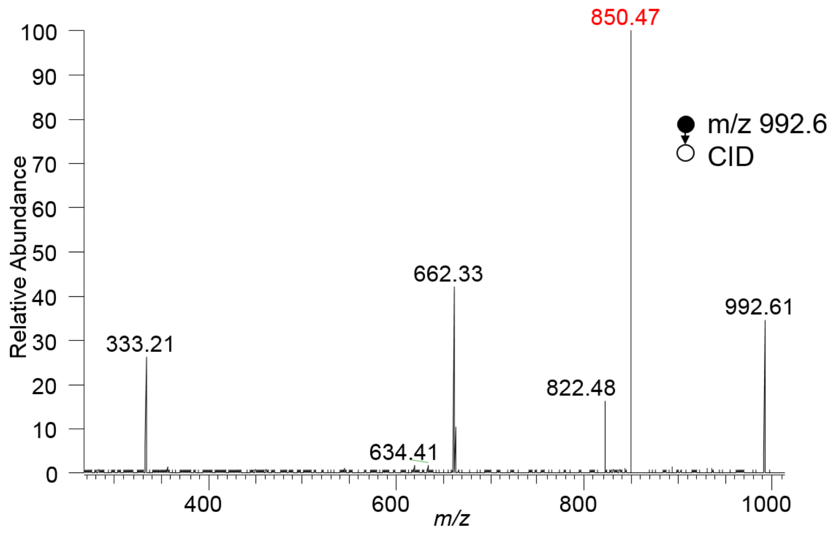

Figure S8. The MS^2 spectrum of derivatized PC 16:0_18:1 (Δ9) (*m/z* 992.6) at 750 nM, producing diagnostic ions *m/z* 850.47 in negative ion mode.

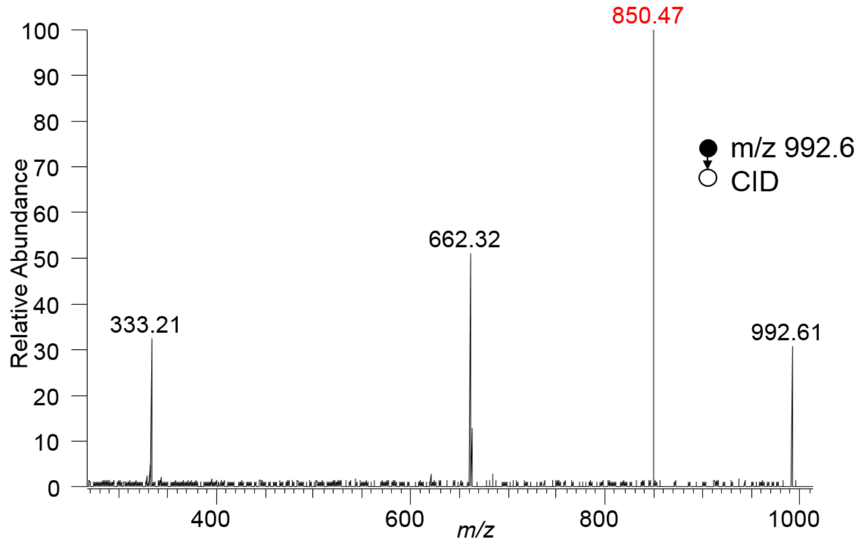

Figure S9. The MS^2 spectrum of derivatized PC 16:0_18:1 (Δ9) (*m/z* 992.6) at 150 nM, producing diagnostic ions *m/z* 850.47 in negative ion mode.

The discussion of the strengths and weaknesses of our method: The strengths and weaknesses of the DNPZ tagging method are summarized here. One of the key advantages of DNPZ tagging is its ability to address the limitations of previous C=C bond derivatization and charge-switching strategies. Approaches that can localize double bond positions in GPLs are typically limited to phosphatidylcholine (PC) species analyzed in positive ion mode and lack the capability for efficient fatty acyl chain identification. Conversely, ion/ion charge

inversion reactions enable analysis of PCs in negative ion mode for acyl chain determination but do not yield diagnostic fragments for C=C bond localization. DNPZ tagging uniquely combines both charge switching and double bond activation in a single workflow, enabling comprehensive structural characterization of PCs and other GPLs in negative ion mode. Additionally, it generates more intense diagnostic ions for C=C localization compared to established methods such as epoxidation or ozonide formation. A limitation of the DNPZ method, however, is its relatively high limit of detection at room temperature (~1 μM), which may restrict its sensitivity for low-abundance lipid species. This sensitivity can be improved by conducting the derivatization at lower temperatures (e.g., –10 °C), where the LOD is reduced to 150 nM.

Section S6. Calibration curve

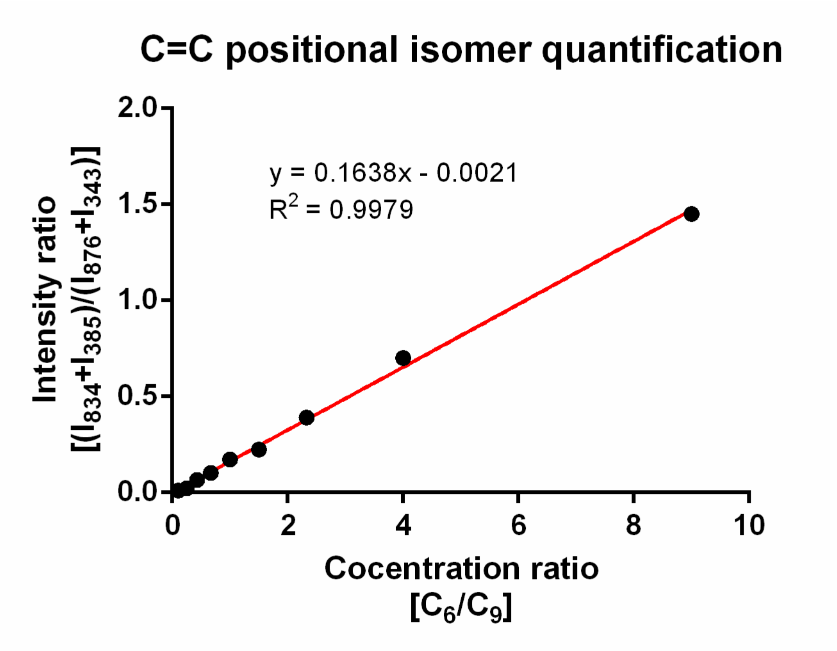

Figure S10. The calibration curve was established to correlate the diagnostic ion intensity ratio ($I_{(834+385)}/I_{(876+343)}$) with the concentration ratio (C_6/C_9) of PC 18:1 positional isomers with carbon-carbon double bonds

Section S7. Proposed structures of aldehyde diagnostic ions

The proposed structures for the aldehyde diagnostic ions are shown in Figure S11. The most likely deprotonated site is the α-proton adjacent to the aldehyde group on the fatty acyl chain. Upon deprotonation, the resulting negative charge forms an enolate, which is stabilized through resonance with the adjacent vinyl groups, providing a plausible explanation for the formation and stability of the aldehyde diagnostic ion observed at *m/z* 632.39 (Δ8), 672.42 (Δ11), and 712.45 (Δ14). However, in the case of the aldehyde diagnostic ion at Δ5, no vinyl group is present to stabilize the negative charge. As a result, only the demethylated aldehyde diagnostic ion at *m/z* 578.34 is observed in the MS^2 spectrum.

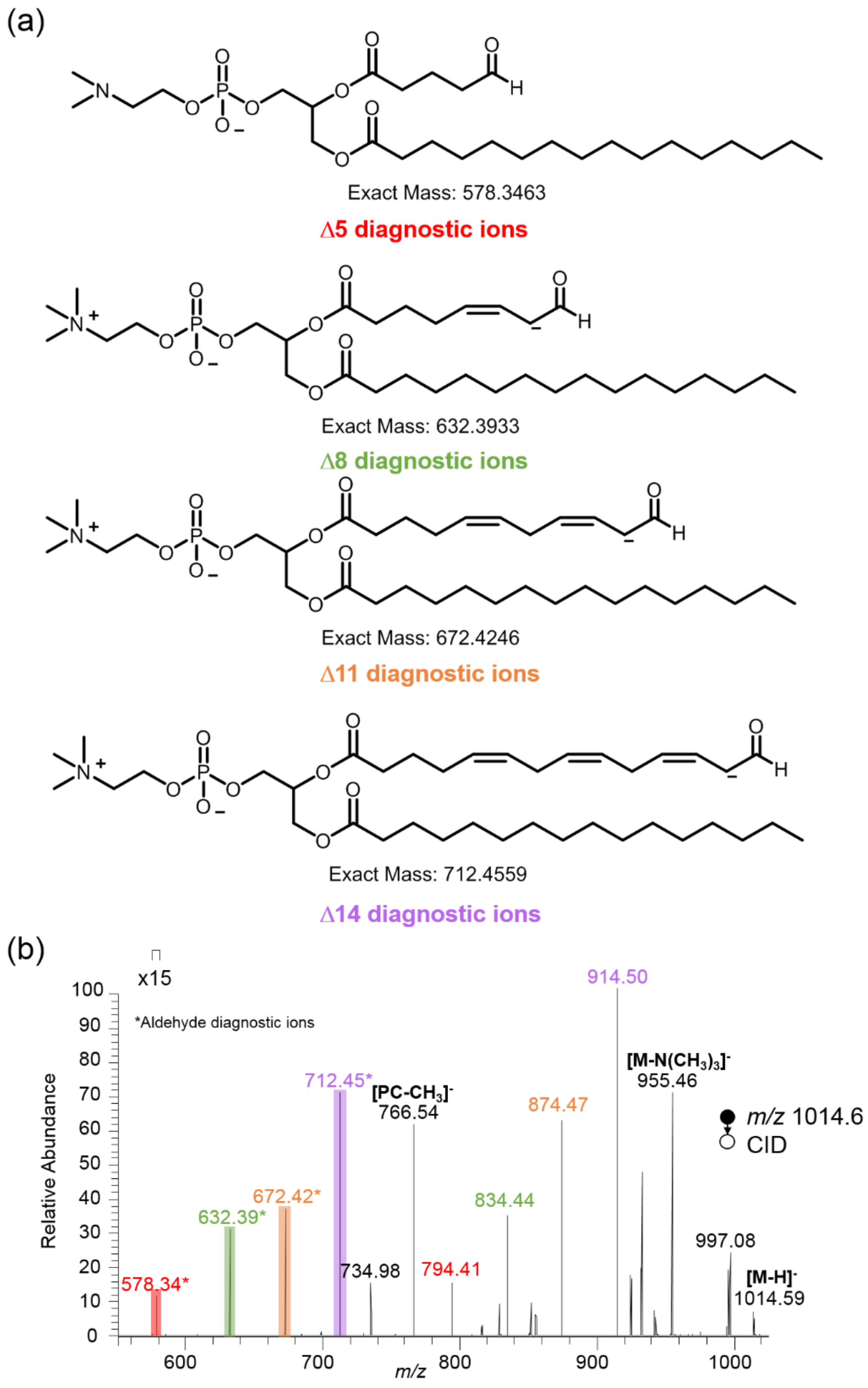

Figure S11. (a) Proposed structures of aldehyde diagnostic ions for PC 16:0_20:4 (Δ5, Δ8, Δ11, Δ14). (b) CID spectrum of derivatized PC 16:0_20:4 (Δ5, Δ8, Δ11, Δ14), showing the aldehyde diagnostic ions at m/z 578.34 (Δ5), 632.39 (Δ8), 672.42 (Δ11), and 712.45 (Δ14).

Section S8. Tandem mass spectra for structural characterization of different GPL subclass standards

We evaluated the reaction efficiency with various GPL subclasses, including PE, PA, PS, and PG, by calculating the ratio of the derivatized product intensity to the native lipid intensity in negative ion mode. The observed efficiencies ranged from 5%-24%.

Structural characterization of of PA 34:1

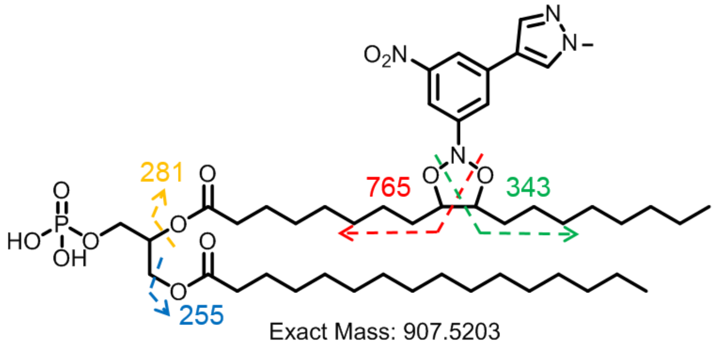

PA 16:0_18:1 (Δ9)

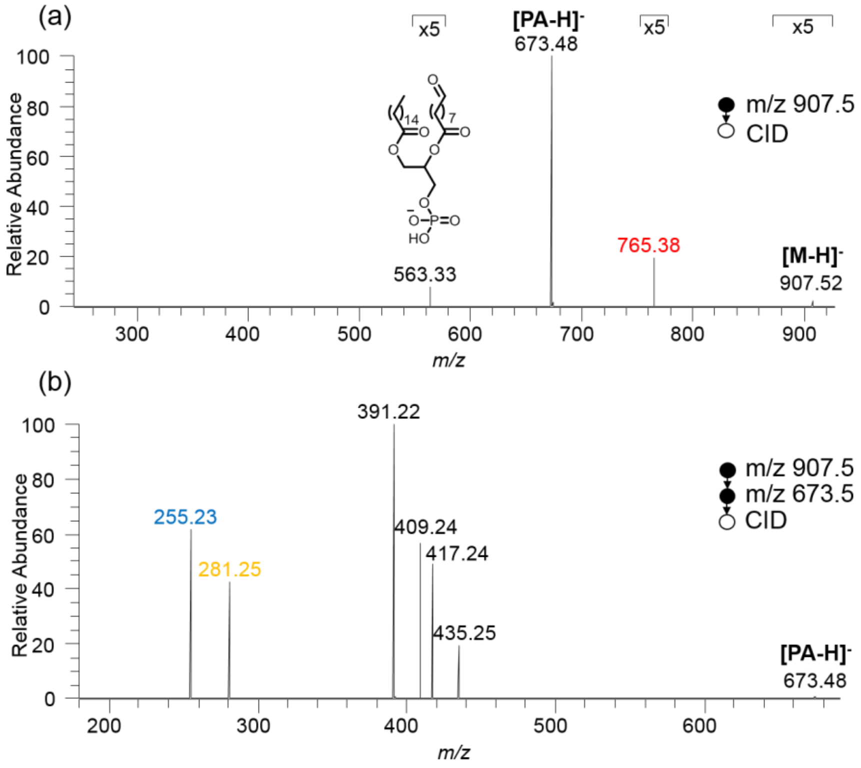

Figure S12. (a) MS^2 spectra of derivatized PA 34:1 at *m/z* 907.5 upon CID fragmentation (b) MS^3 spectra of deprotonated PA 34:1 at *m/z* 673.5 upon CID fragmentation. PA 34:1 can then be identified as PA 16:0_18:1 (Δ9). The conversion ratio of PA derivatization is 23.7%.

Structural characterization of of PG 34:1

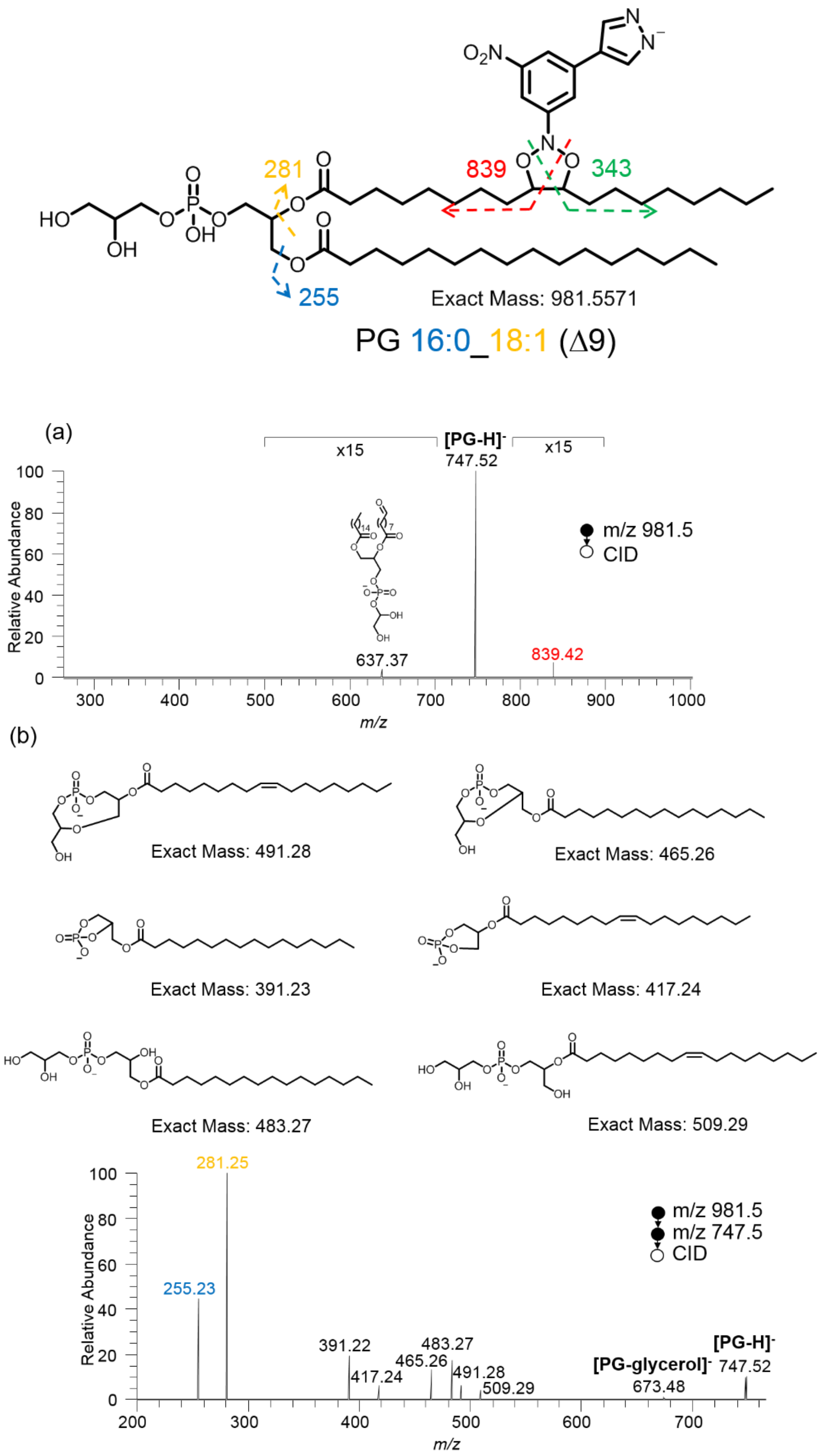

Figure S13. (a) MS^2 spectra of derivatized PG 34:1 at *m/z* 981.5 upon CID fragmentation (b) MS^3 spectra of deprotonated PG 34:1 at *m/z* 747.5 upon CID fragmentation. PG 34:1 can then be identified as PG 16:0_18:1 (Δ9). The conversion ratio of PG derivatization is 4.5%.

Structural characterization of of PS 34:1

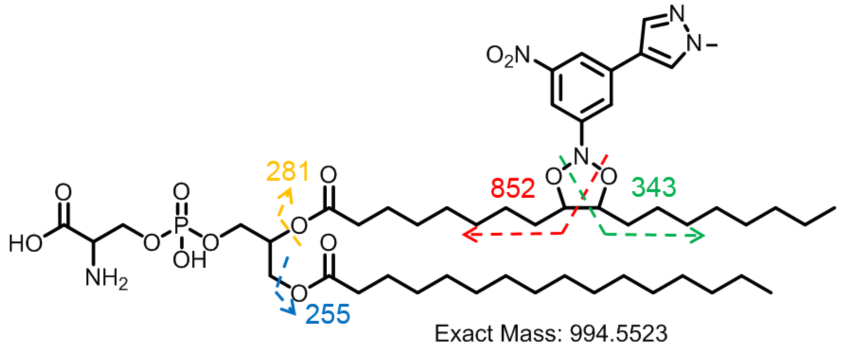

PS 16:0_18:1 (Δ9)

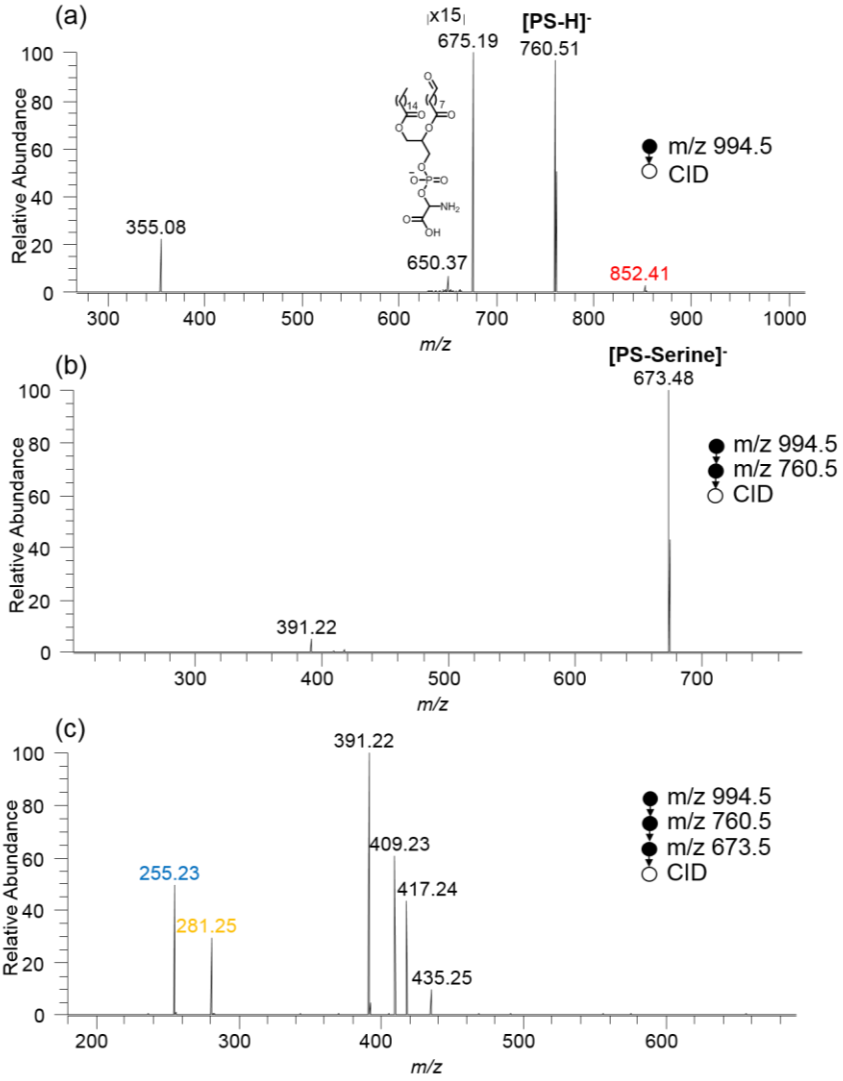

Figure S14. (a) MS^2 spectra of derivatized PS 34:1 at *m/z* 994.5 upon CID fragmentation (b) MS^3 spectra of deprotonated PS 34:1 at *m/z* 760.5 upon CID fragmentation. (b) MS^4 spectra of deprotonated PS 34:1 at *m/z* 673.5 upon CID fragmentation. PS 34:1 can then be identified as PS 16:0_18:1 (Δ9). The conversion ratio of PS derivatization is 8.5%.

Structural characterization of of PE 34:1

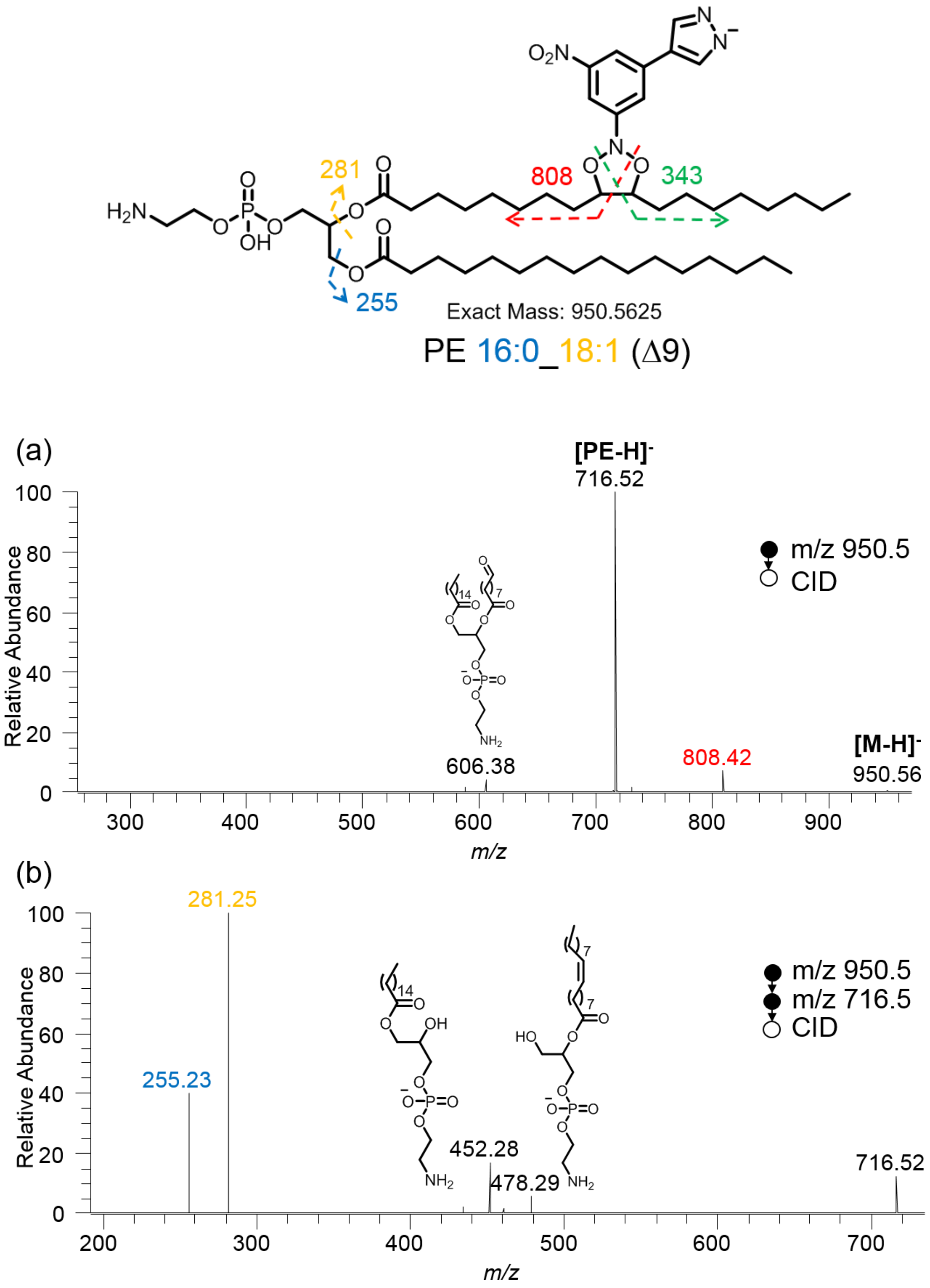

Figure S15. (a) MS^2 spectra of derivatized PE 34:1 at *m/z* 950.5 upon CID fragmentation (b) MS^3 spectra of deprotonated PE 34:1 at *m/z* 716.5 upon CID fragmentation. PE 34:1 can then be identified as PE 16:0_18:1 (Δ9). The conversion ratio of PE derivatization is 4.5%.

Section S9. Tandem mass spectra for structural characterization of glycerophospholipids in soybean polar lipid extracts

Structural characterization of PC 34:2

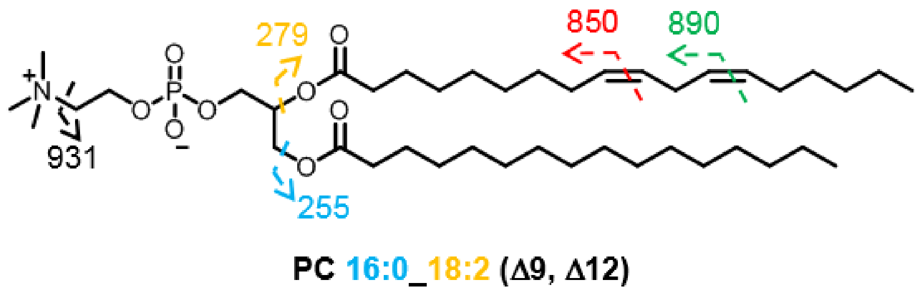

(a) CID fragmentation of deprotonated ions at *m/z* 990.7

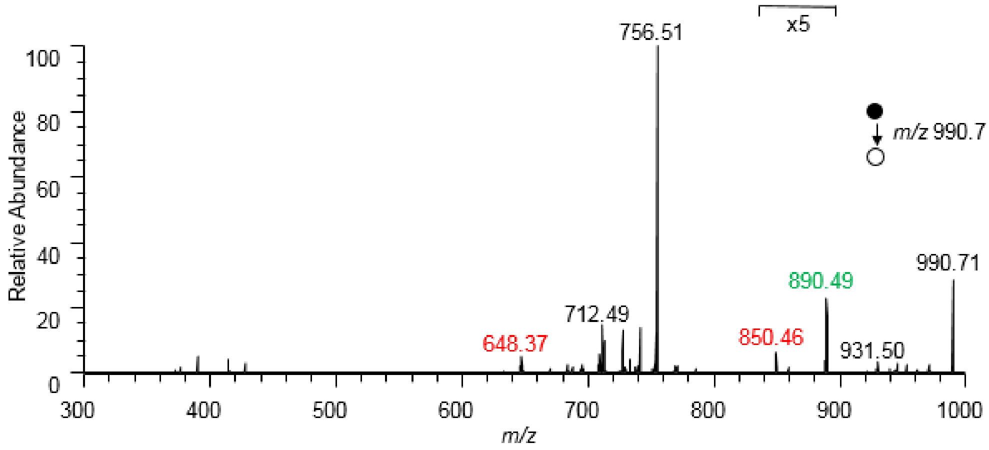

(b) CID fragmentation of deprotonated ions at *m/z* 756.5

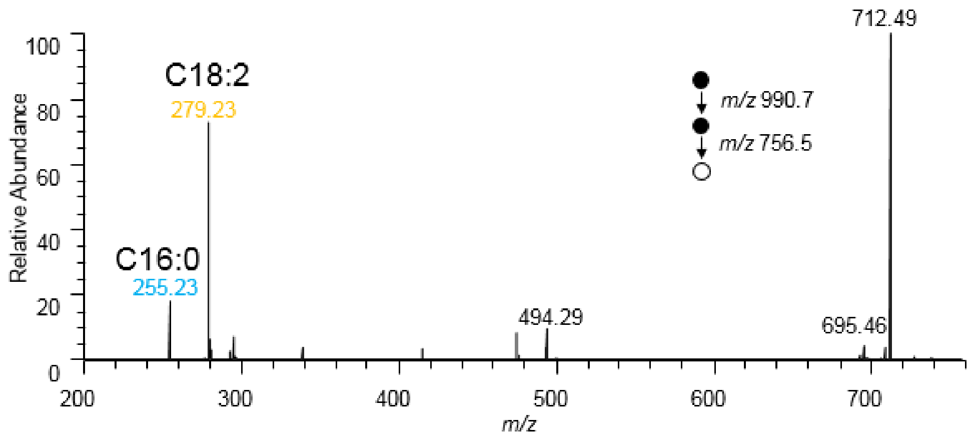

Figure S16. (a) MS^2 spectra of derivatized PC 34:2 at *m/z* 990.7 upon CID fragmentation (b) MS^3 spectra of deprotonated PC 34:2 at *m/z* 756.5 upon CID fragmentation. PC 34:2 can then be identified as PC 16:0_18:2 (Δ9, Δ12).

Structural characterization of PC 34:3

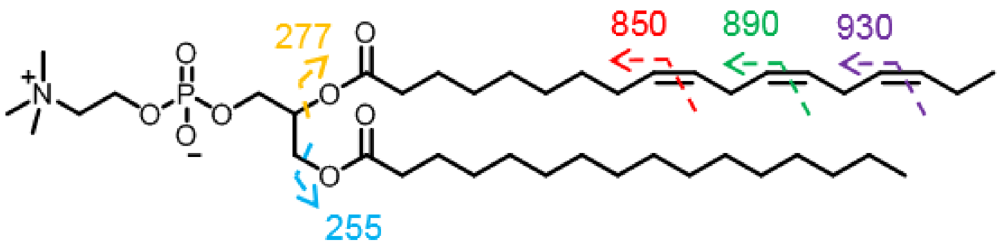

PC 16:0_18:3(Δ9, Δ12, Δ15)

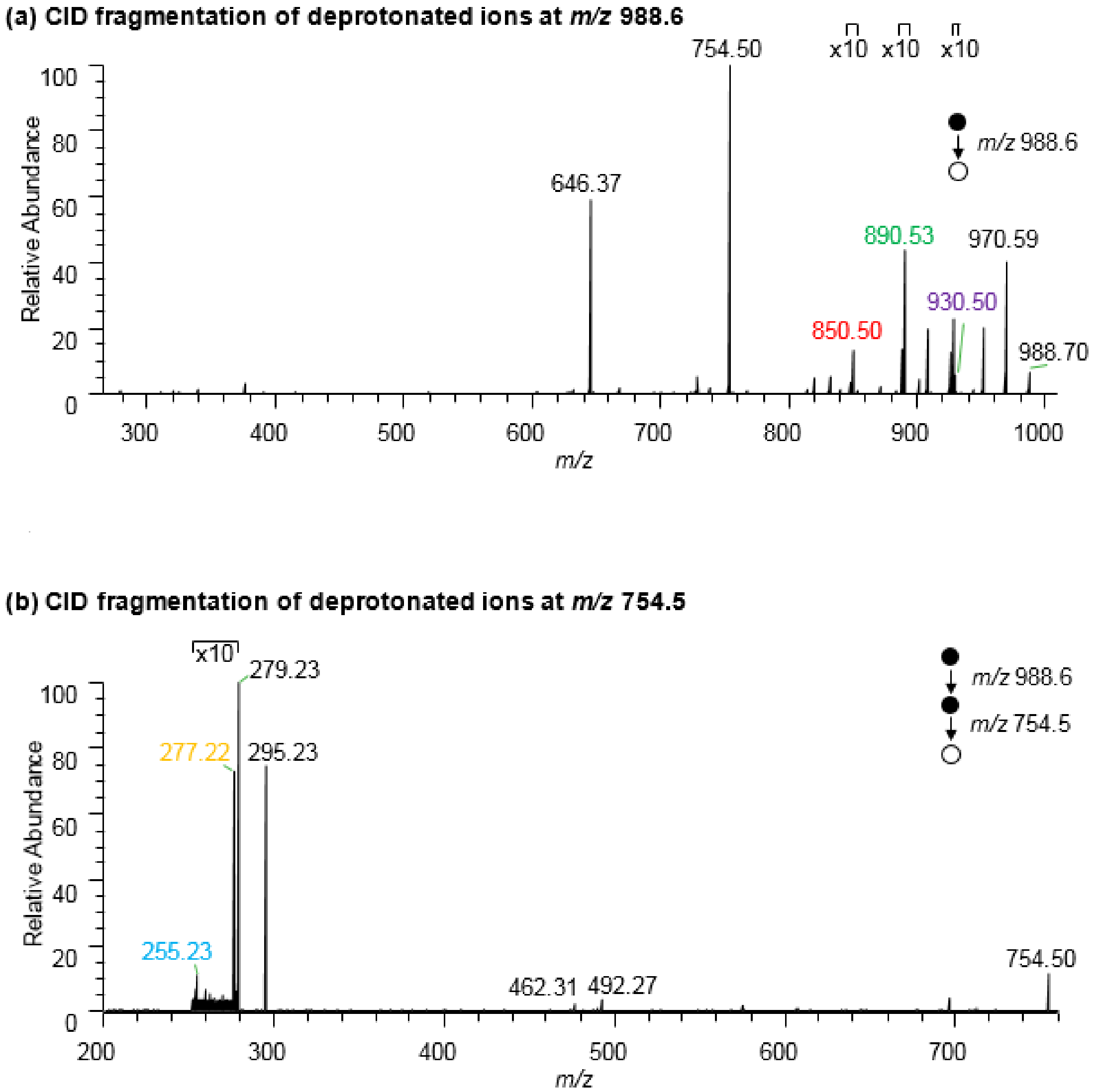

Figure S17. (a) MS^2 spectra of derivatized PC 34:3 at *m/z* 988.6 upon CID fragmentation (b) MS^3 spectra of deprotonated PC 34:3 at *m/z* 754.5 upon CID fragmentation. PC 34:3 can then be identified as PC 16:0_18:3 (Δ9, Δ12, Δ15).

Structural characterization of PC 36:4

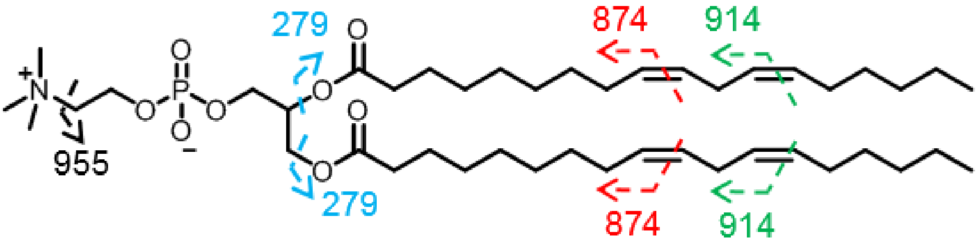

PC 18:2(Δ9, Δ12)_18:2(Δ9, Δ12)

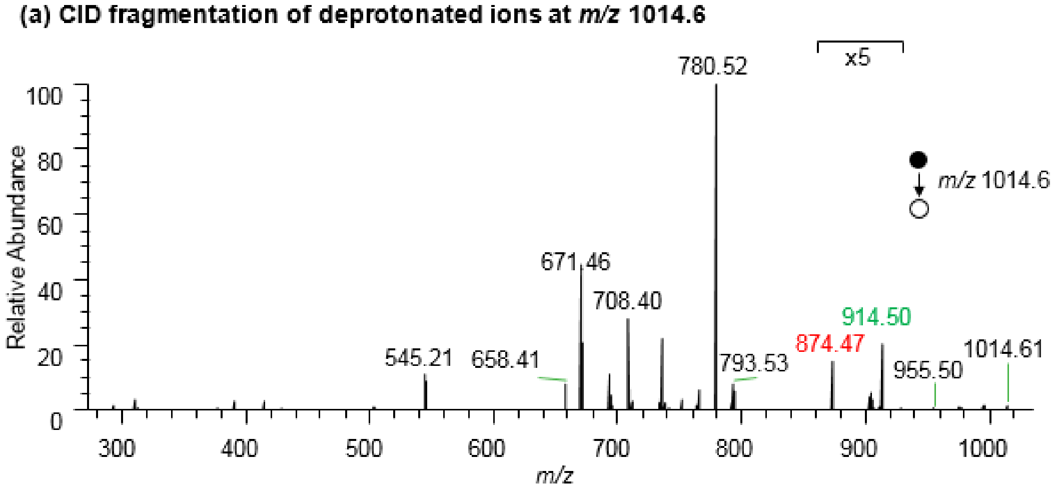

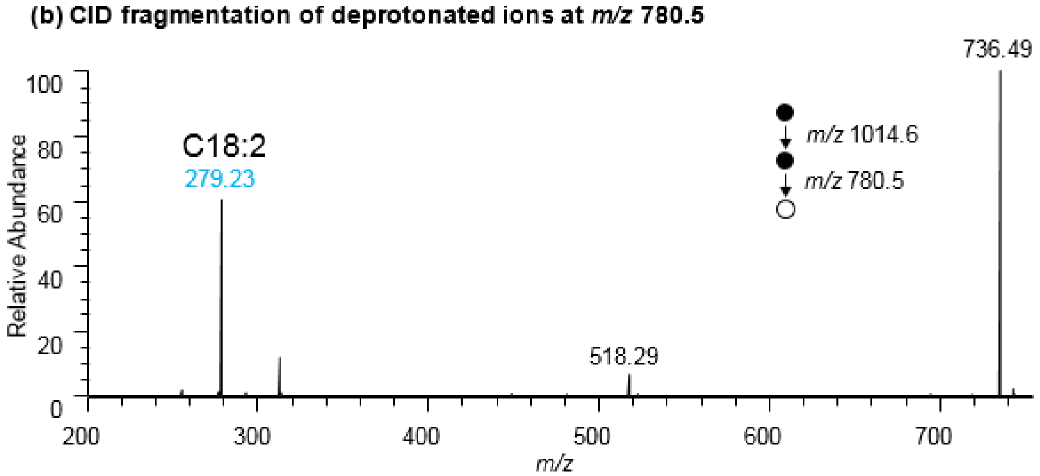

Figure S18. (a) MS^2 spectra of derivatized PC 36:4 at *m/z* 1014.6 upon CID fragmentation (b) MS^3 spectra of deprotonated PC 36:4 at *m/z* 780.5 upon CID fragmentation. PC 36:4 can then be identified as PC 18:2 (Δ9, Δ12)_18:2 (Δ9, Δ12).

Structural characterization of PC 36:5

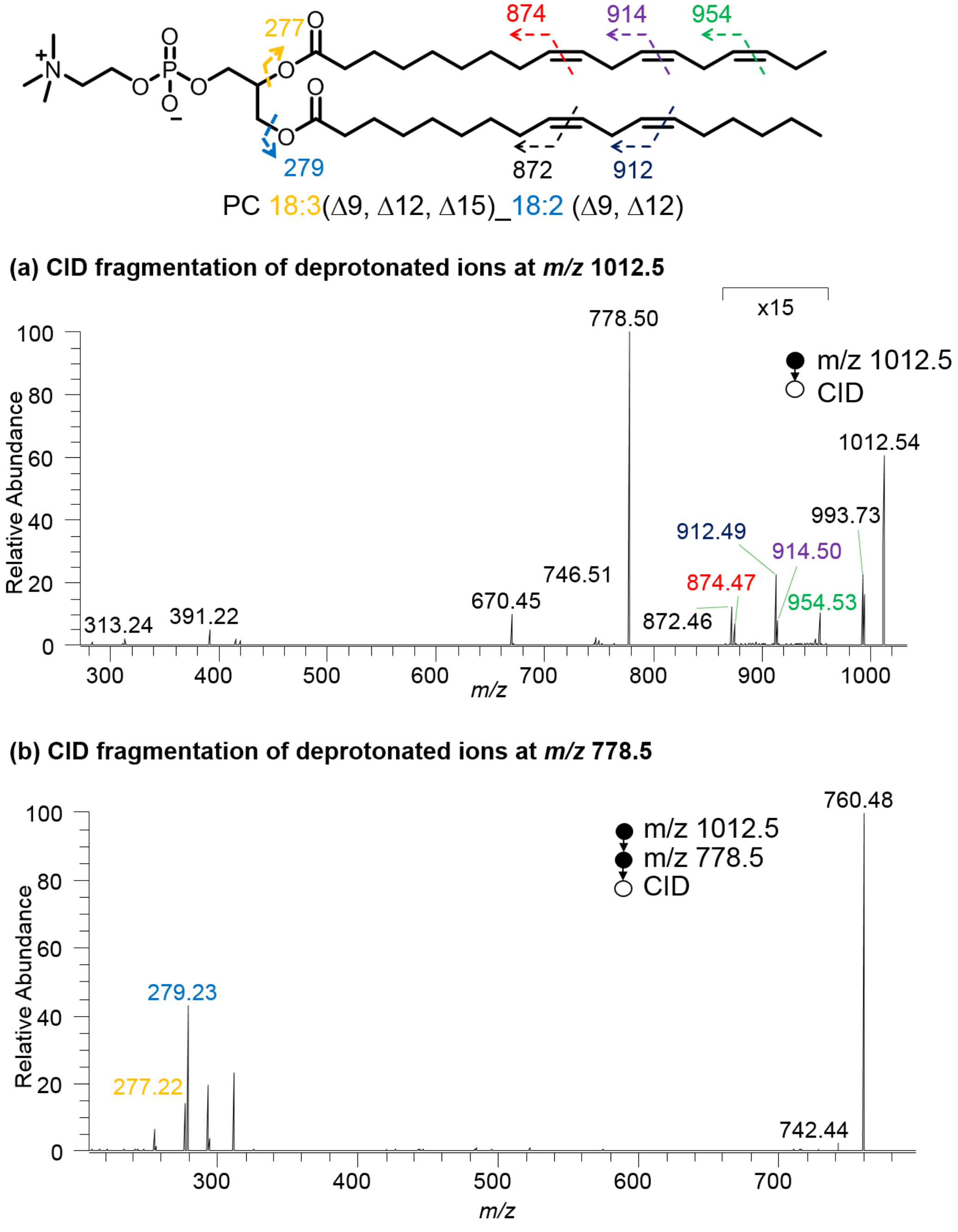

Figure S19. (a) MS^2 spectra of derivatized PC 36:5 at *m/z* 1012.5 upon CID fragmentation (b) MS^3 spectra of deprotonated PC 36:5 at *m/z* 778.5 upon CID fragmentation. PC 36:5 can then be identified as PC 18:3 (Δ9, Δ12, Δ15)_18:2 (Δ9, Δ12).

Structural characterization of PE 34:2

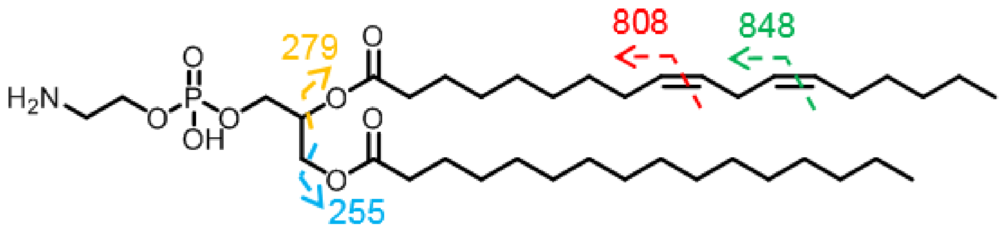

PE 16:0_18:2(Δ9, Δ12)

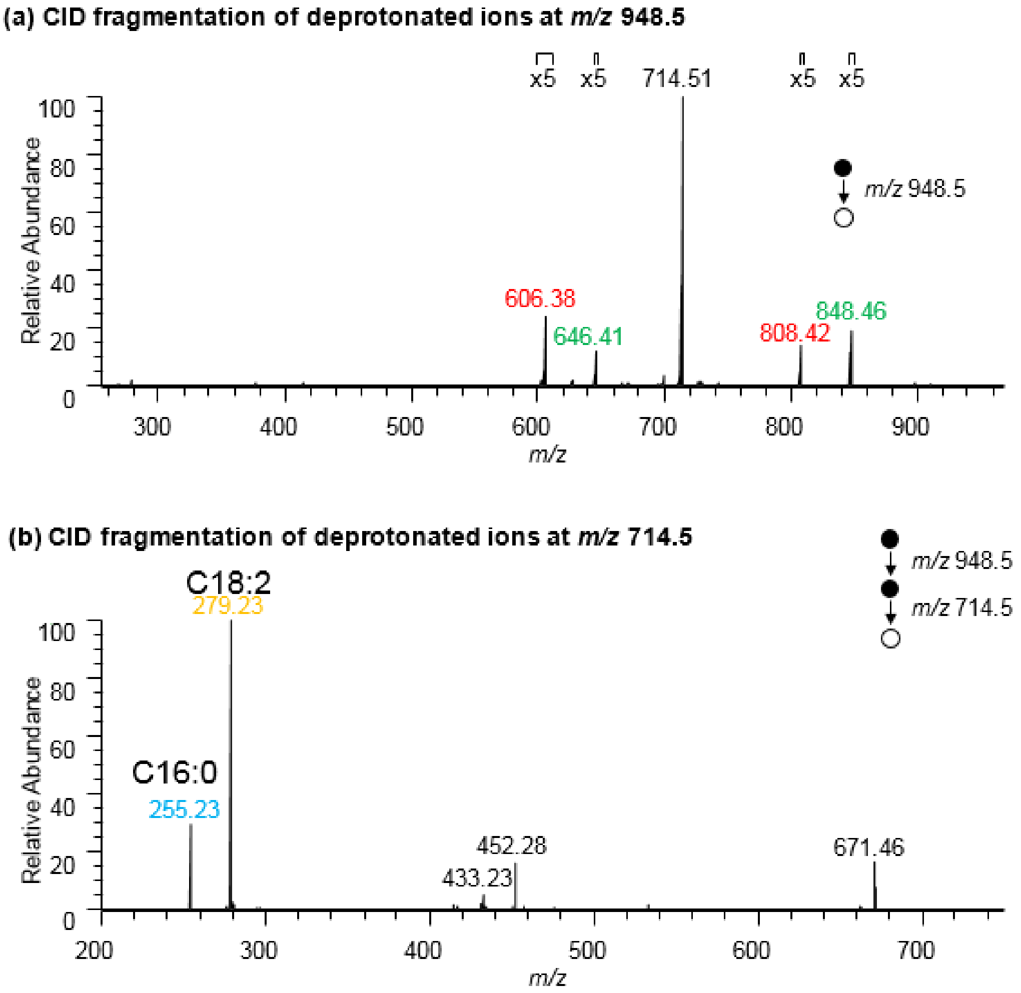

Figure S20. (a) MS^2 spectra of derivatized PE 34:2 at *m/z* 948.5 upon CID fragmentation (b) MS^3 spectra of deprotonated PE 34:2 at *m/z* 714.51 upon CID fragmentation. PE 34:2 can then be identified as PE 16:0_18:2 (Δ9, Δ12).

Structural characterization of PE 36:4

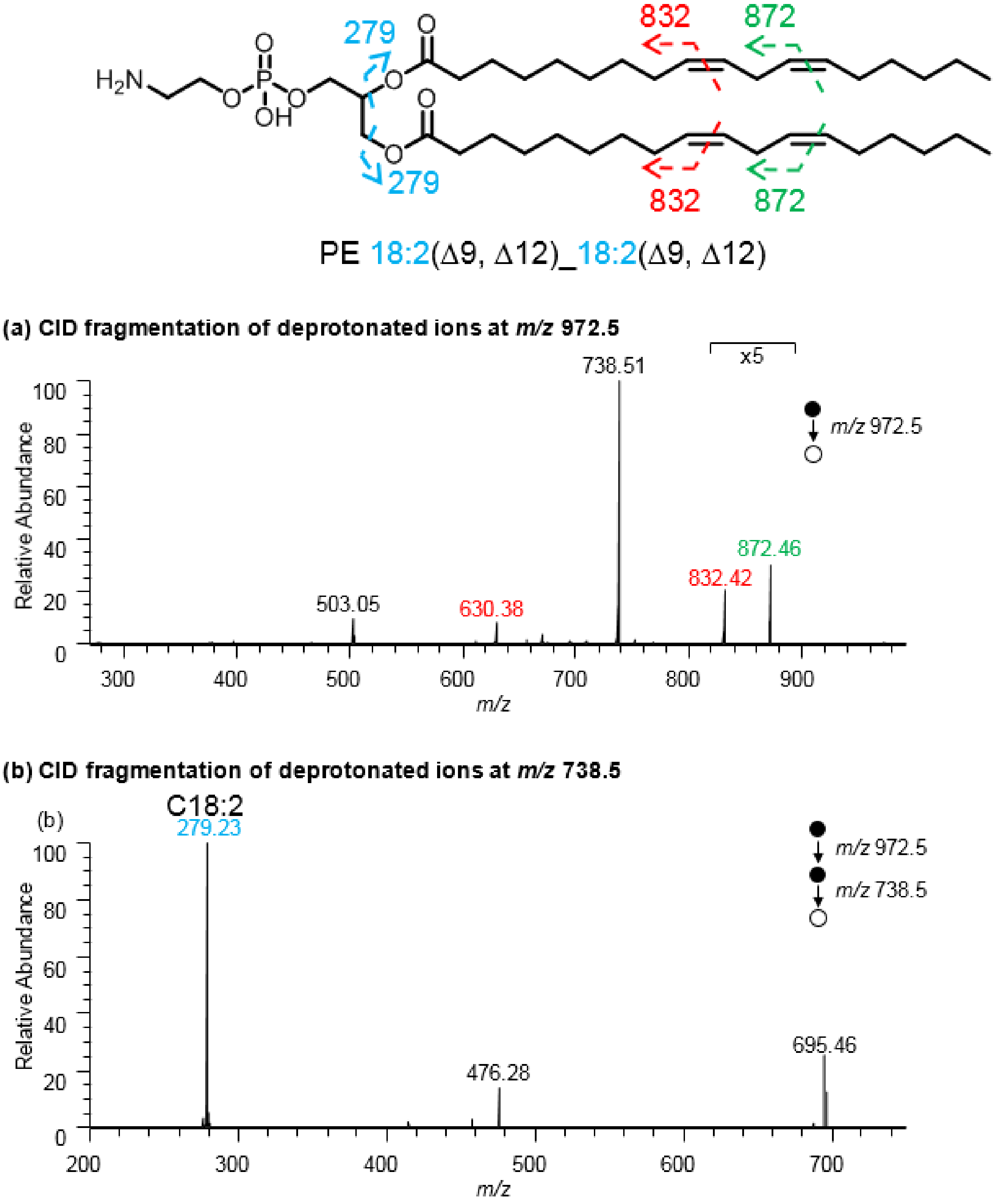

Figure S21. (a) MS^2 spectra of derivatized PE 36:4 at *m/z* 972.5 upon CID fragmentation (b) MS^3 spectra of deprotonated PE 36:4 at *m/z* 738.5 upon CID fragmentation. PE 36:4 can then be identified as PE 18:2 (Δ9, Δ12)_18:2 (Δ9, Δ12).

Structural characterization of PE 36:5

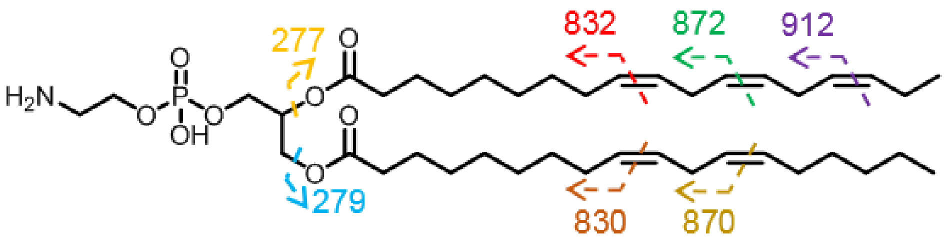

PE 18:3(Δ9, Δ12, Δ15)_18:2(Δ9, Δ12)

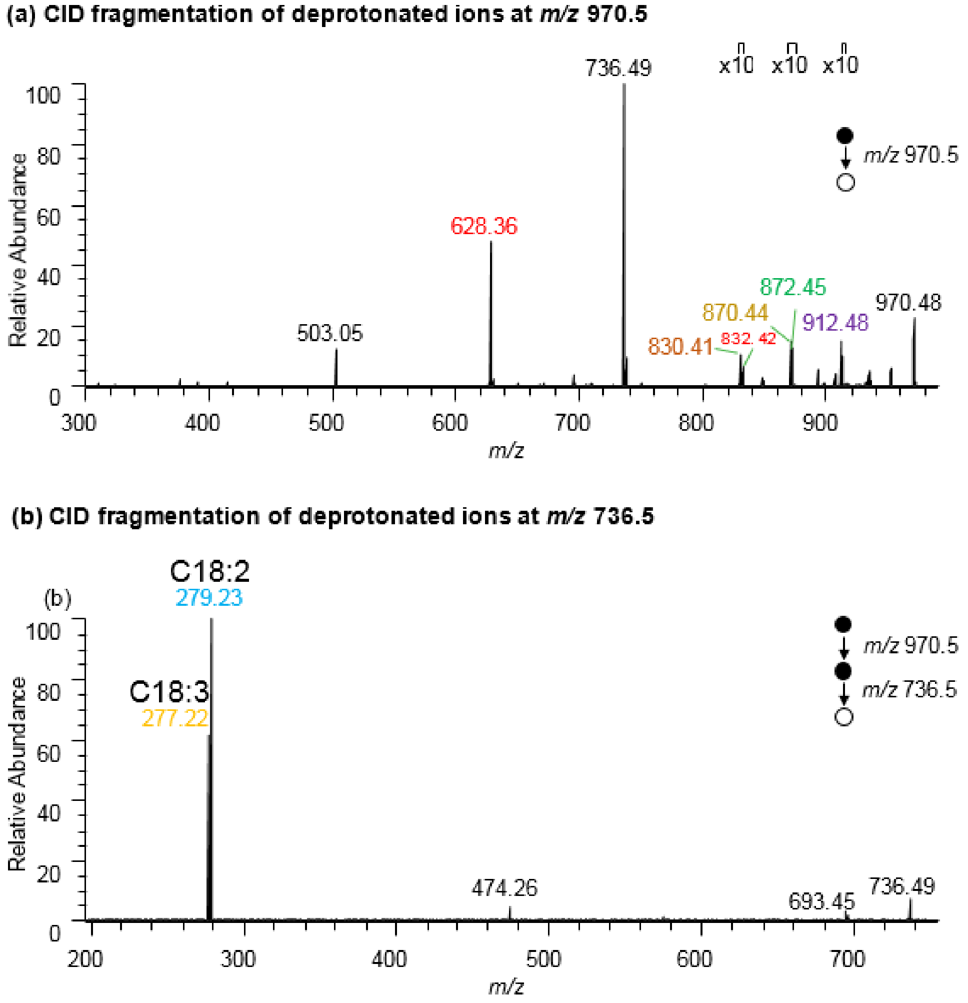

Figure S22. (a) MS^2 spectra of derivatized PE 36:5 at *m/z* 970.5 upon CID fragmentation (b) MS^3 spectra of deprotonated PE 36:5 at *m/z* 736.5 upon CID fragmentation. PE 36:5 can then be identified as PE 18:3 (Δ9, Δ12, Δ15)_18:2 (Δ9, Δ12).

Structural characterization of PE 37:3

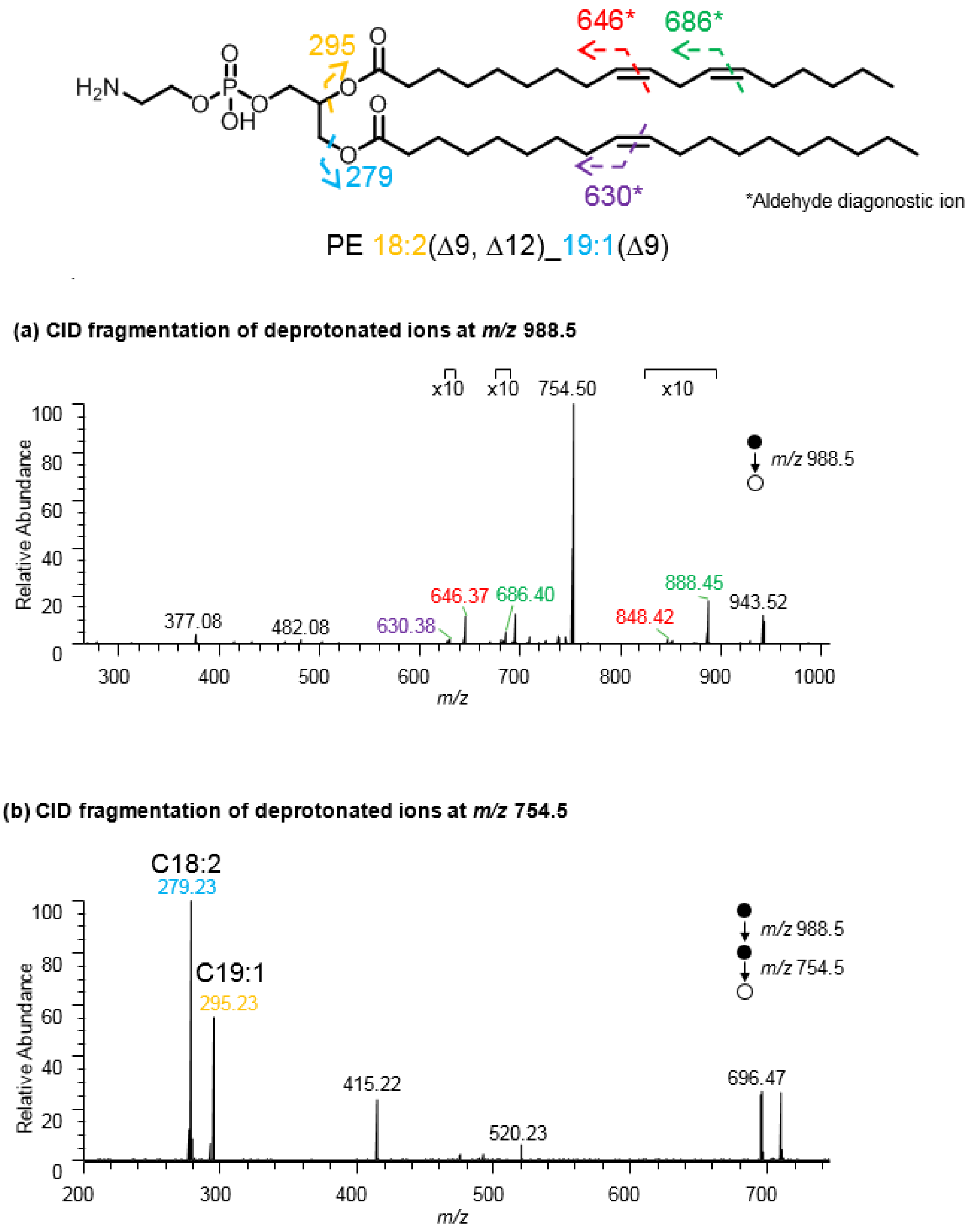

Figure S23. (a) MS^2 spectra of derivatized PE 37:3 at *m/z* 988.5 upon CID fragmentation (b) MS^3 spectra of deprotonated PE 37:3 at *m/z* 754.5 upon CID fragmentation. PE 37:3 can then be identified as PE 18:2 (Δ9, Δ12)_19:1 (Δ9).

Structural characterization of LysoPI 16:0

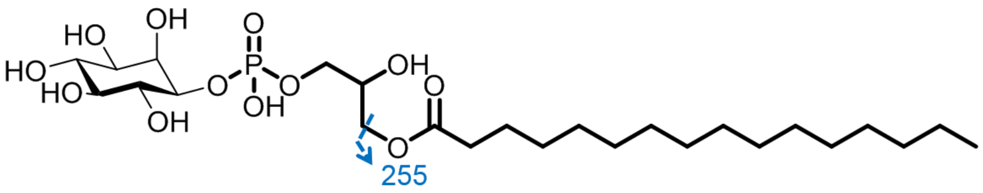

Lyso PI 16:0

(a) CID fragmentation of deprotonated ions at *m/z* 571.3

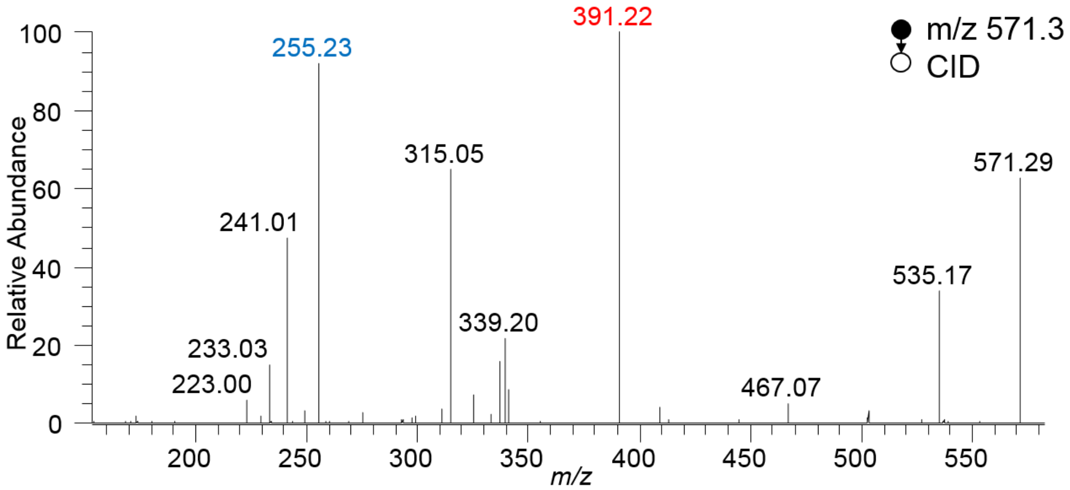

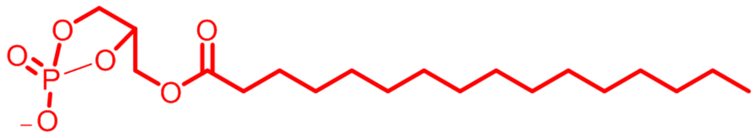

Exact Mass: 391.23

Figure S24. (a) MS^2 spectra of LysoPI 16:0 at *m/z* 571.3 upon CID fragmentation

Structural characterization of PI 34:2

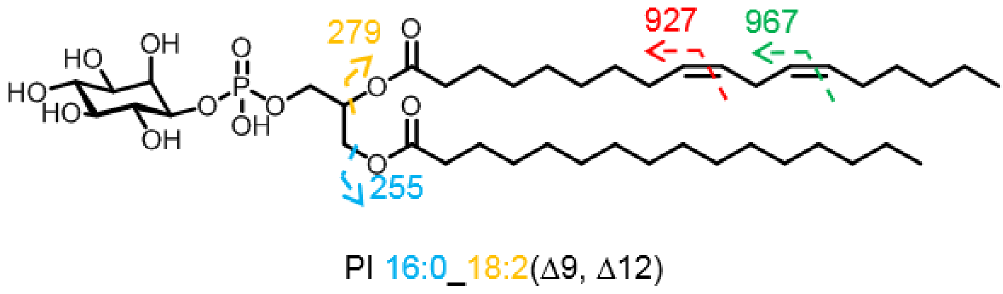

(a) CID fragmentation of deprotonated ions at *m/z* 1067.5

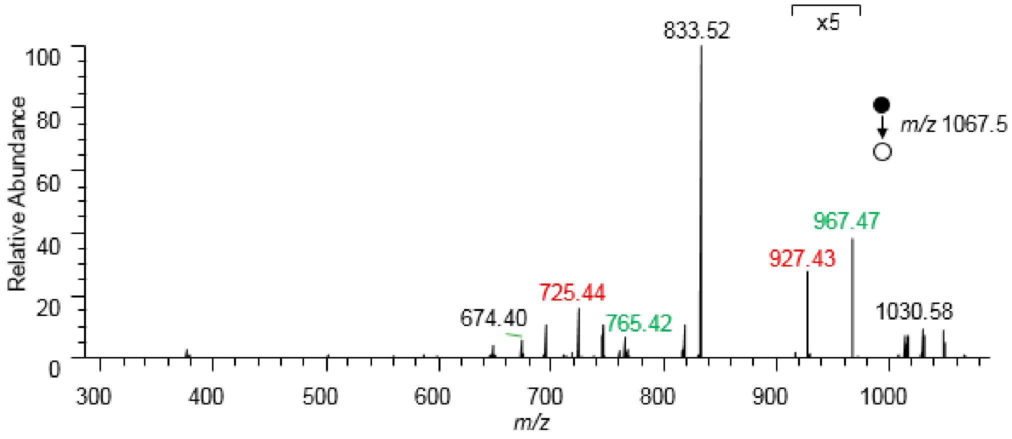

(b) CID fragmentation of deprotonated ions at *m/z* 833.5

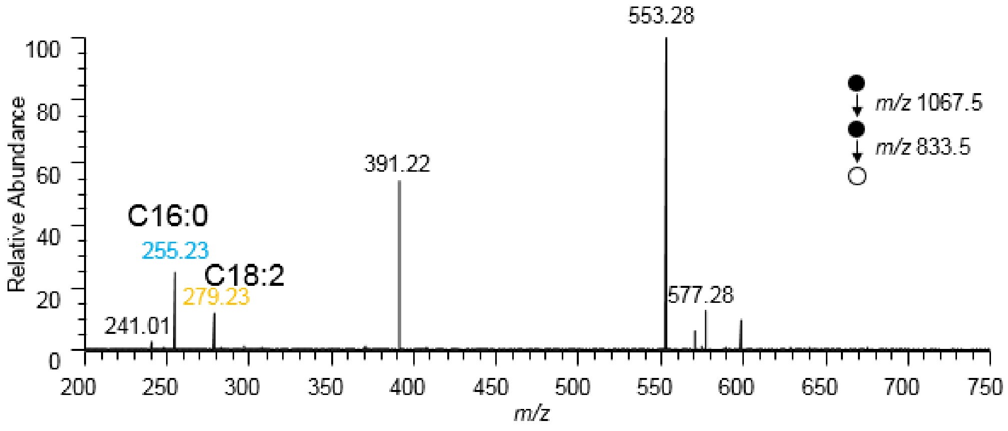

Figure S25. (a) MS^2 spectra of derivatized PI 34:2 at *m/z* 1067.5 upon CID fragmentation (b) MS^3 spectra of deprotonated PI 34:2 at *m/z* 833.5 upon CID fragmentation. PI 34:2 can then be identified as PI 16:0_18:2 (Δ9, Δ12).

Structural characterization of PI 34:3

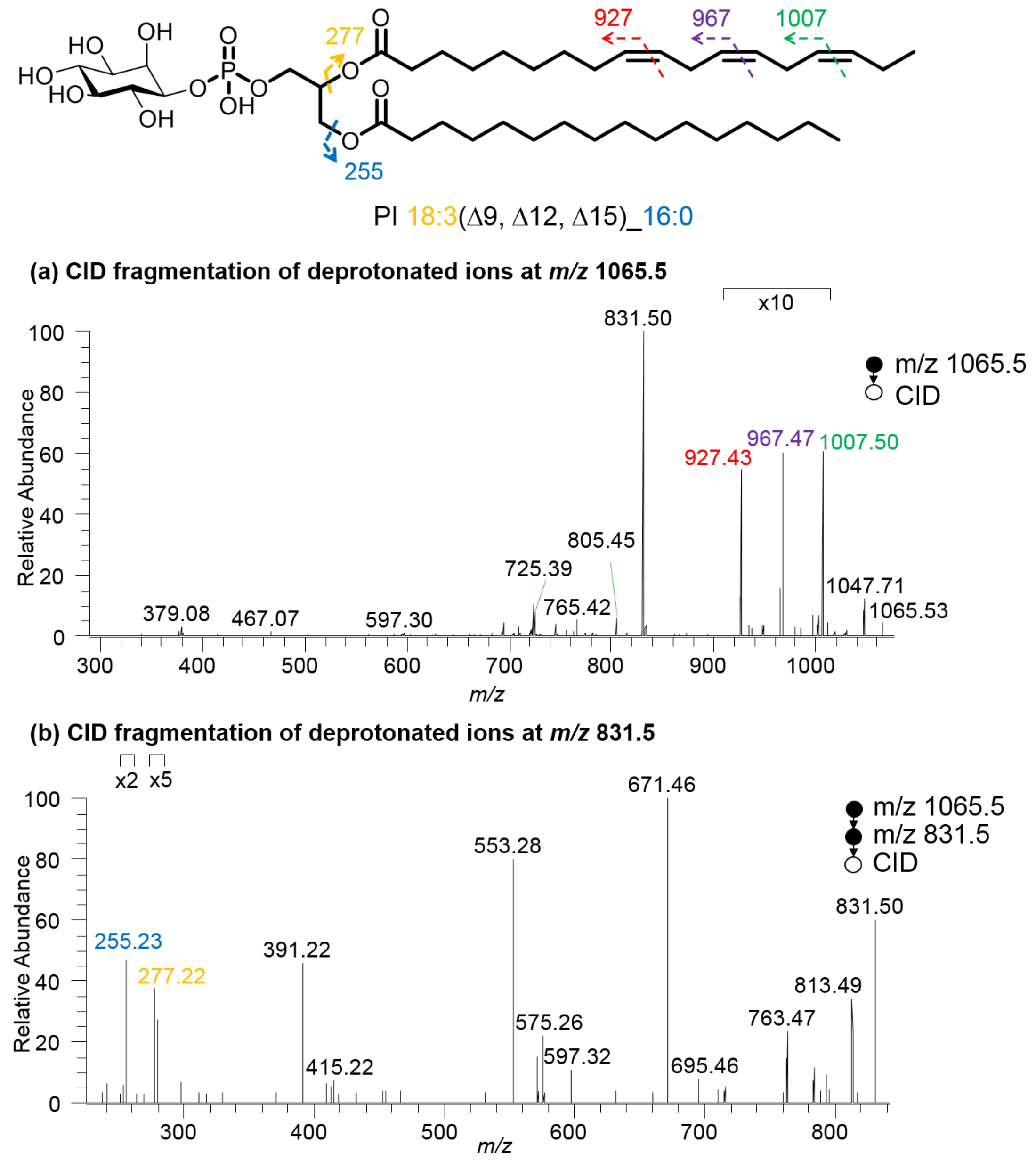

Figure S26. (a) MS^2 spectra of derivatized PI 34:3 at *m/z* 1065.5 upon CID fragmentation (b) MS^3 spectra of deprotonated PI 34:3 at *m/z* 831.5 upon CID fragmentation. PI 34:3 can then be identified as PI 16:0_18:3 (Δ9, Δ12, Δ15).

Structural characterization of PI 36:4

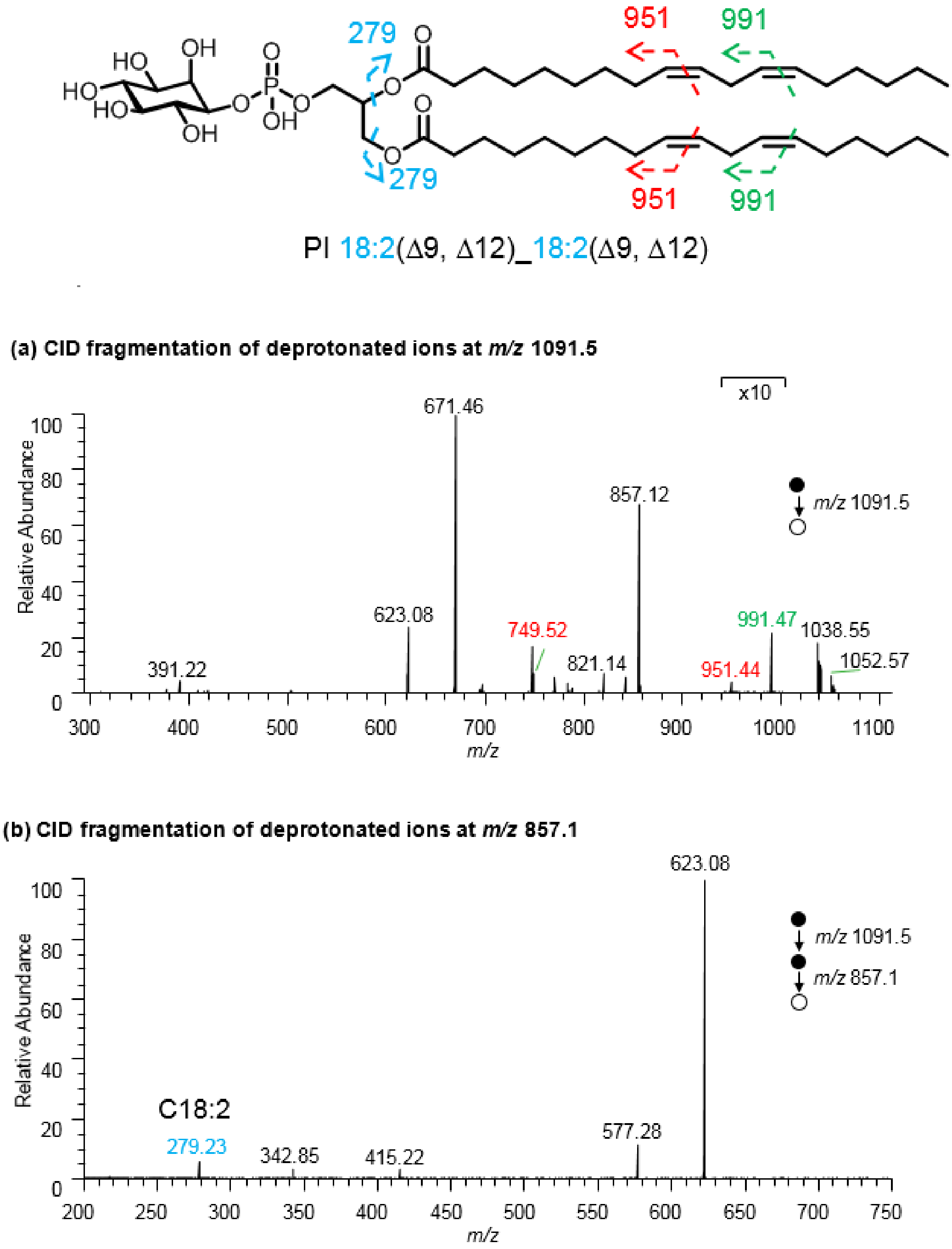

Figure S27. (a) MS^2 spectra of derivatized PI 36:4 at *m/z* 1091.5 upon CID fragmentation (b) MS^3 spectra of deprotonated PI 36:4 at *m/z* 857.1 upon CID fragmentation. PI 36:4 can then be identified as PI 18:2 (Δ9, Δ12)_18:2 (Δ9, Δ12).

Structural characterization of PA 34:2

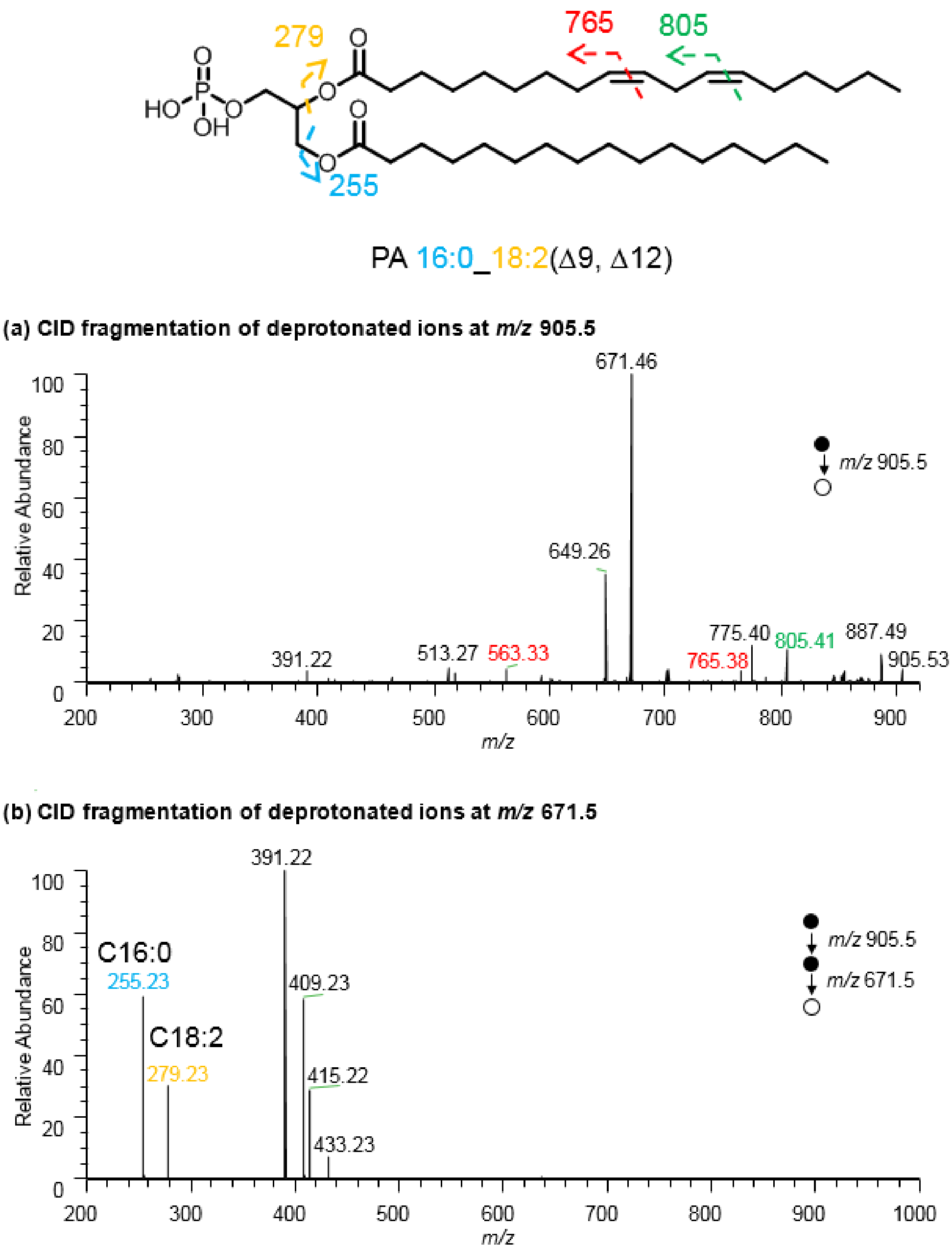

Figure S28. (a) MS^2 spectra of derivatized PA 34:2 at *m/z* 905.5 upon CID fragmentation (b) MS^3 spectra of deprotonated PA 34:2 at *m/z* 671.5 upon CID fragmentation. PA 34:2 can then be identified as PA 16:0_18:2 (Δ9, Δ12).

Structural characterization of PA 34:3

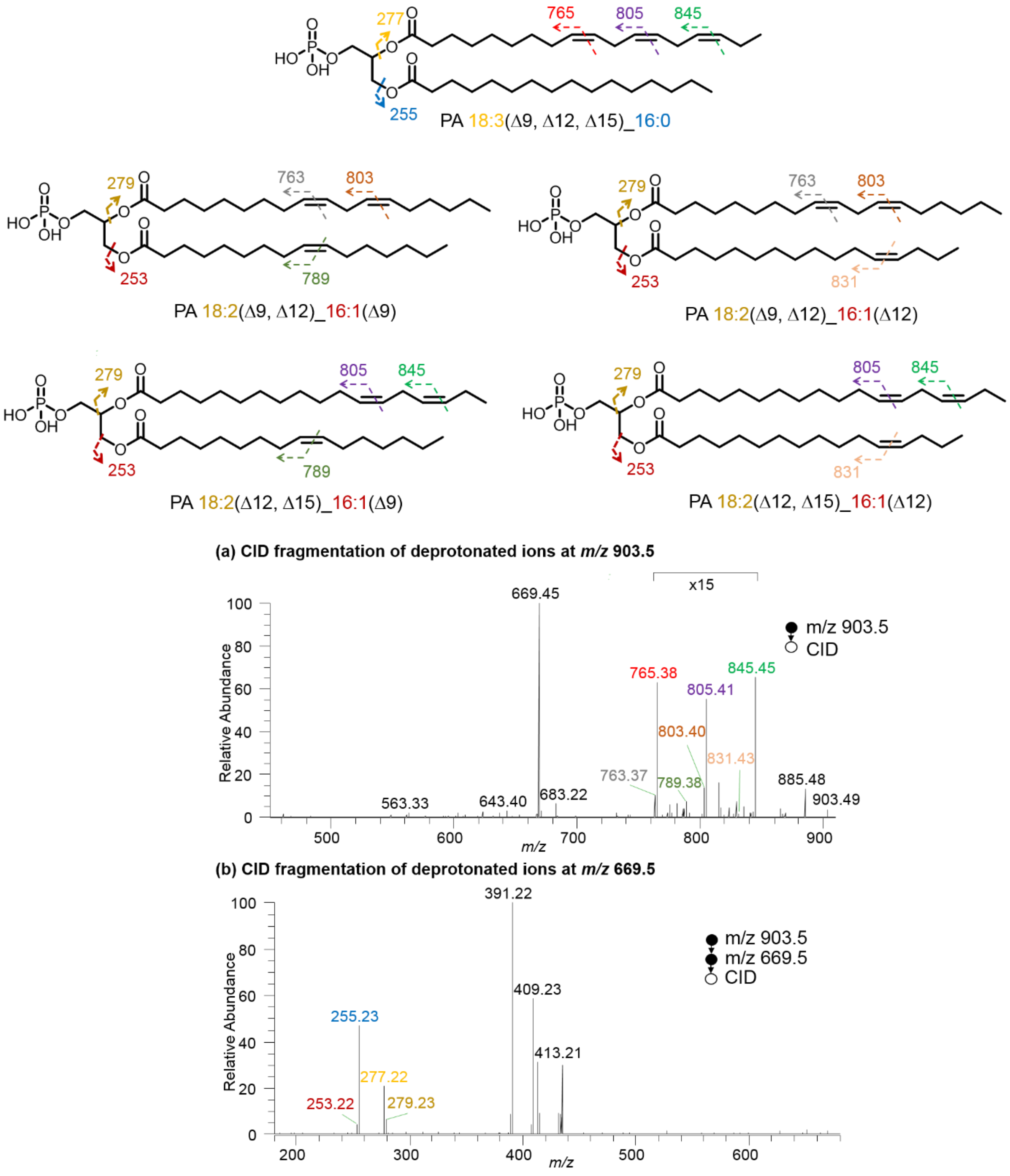

Figure S29. (a) MS^2 spectra of derivatized PA 34:3 at *m/z* 903.5 upon CID fragmentation (b) MS^3 spectra of deprotonated PA 34:3 at *m/z* 669.5 upon CID fragmentation. PA 34:3 can then be identified as PA 18:3(• 9, Δ12, Δ15)_16:0, PA 18:2(Δ9, Δ12)_16:1(Δ9), PA 18:2(Δ9, Δ12)_16:1(Δ12), PA 18:2(Δ12, Δ15)_16:1(Δ9) and PA 18:2(Δ12, Δ15)_16:1(Δ12)

Structural characterization of PA 36:4

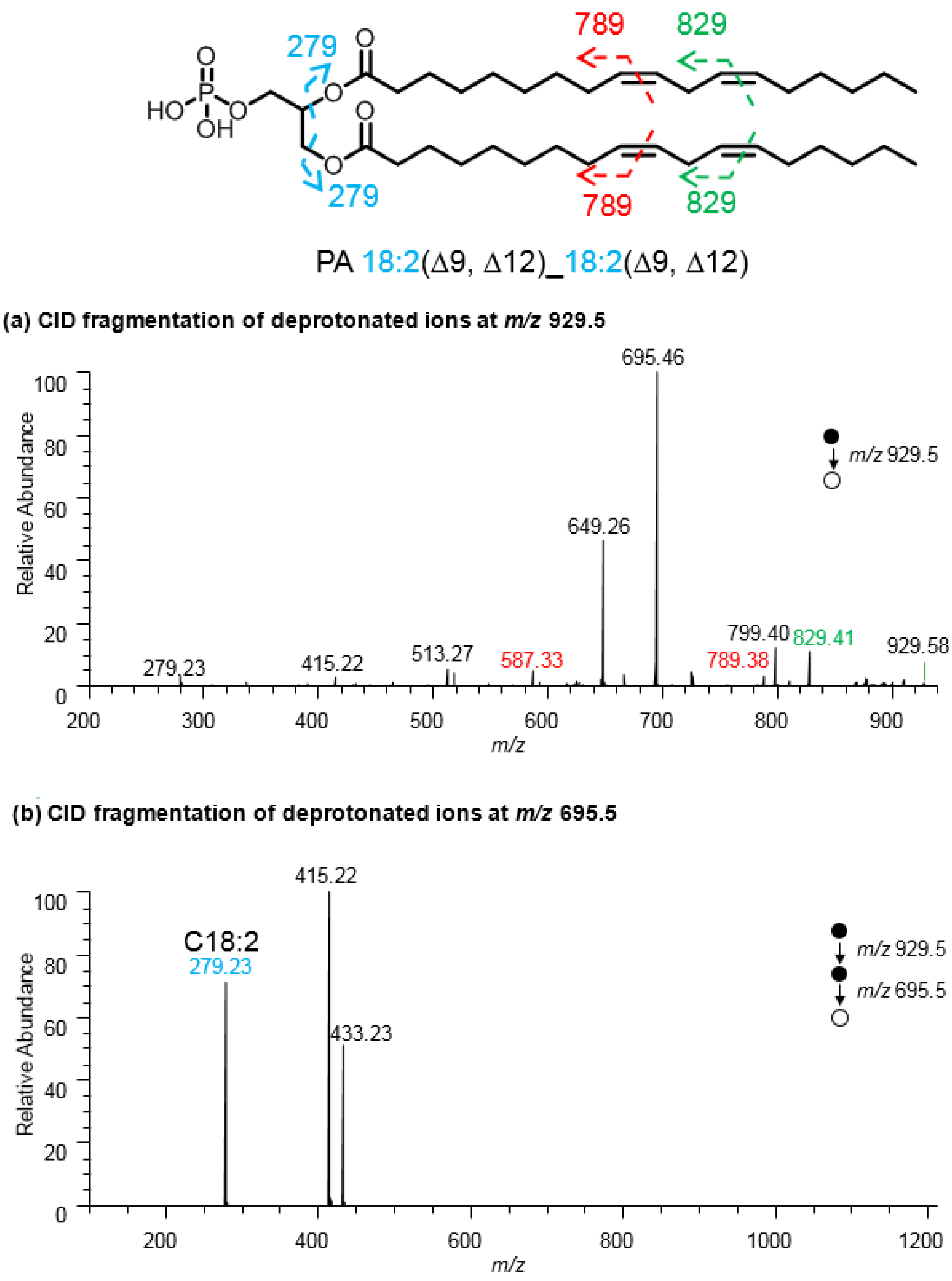

Figure S30. (a) MS^2 spectra of derivatized PA 36:4 at *m/z* 929.5 upon CID fragmentation (b) MS^3 spectra of deprotonated PA 36:4 at *m/z* 695.5 upon CID fragmentation. PA 36:4 can then be identified as PA 18:2 (Δ9, Δ12)_18:2 (Δ9, Δ12).

Structural characterization of PA 36:5

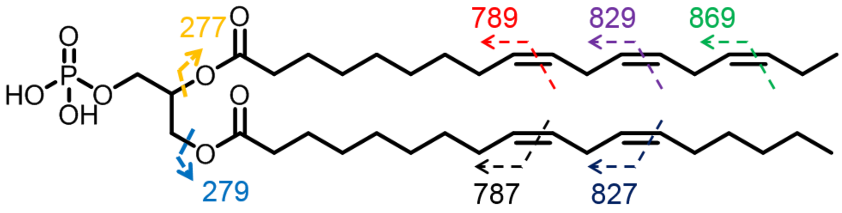

PA 18:3(Δ9, Δ12, Δ15)_18:2 (Δ9, Δ12)

(a) CID fragmentation of deprotonated ions at *m/z* 927.5

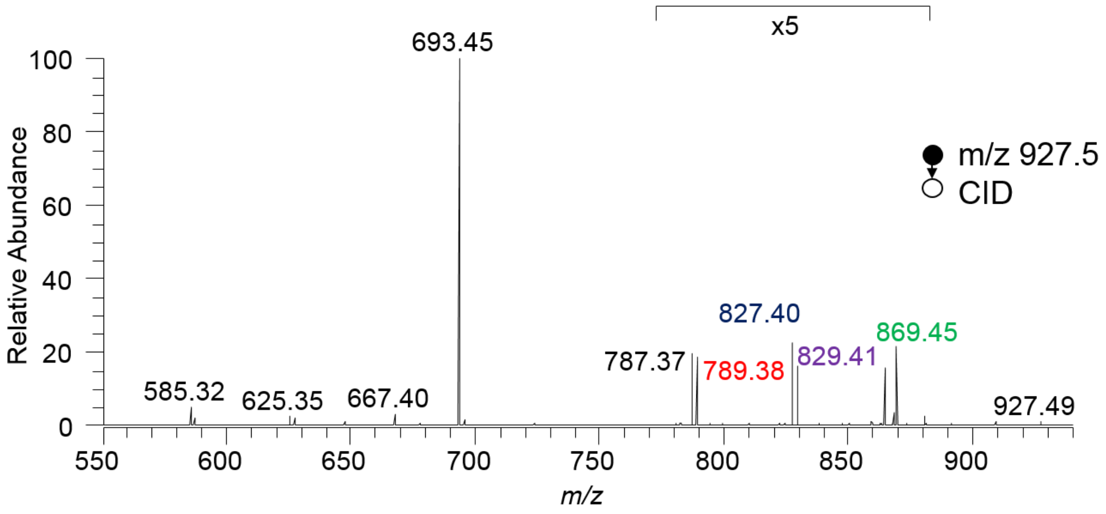

(b) CID fragmentation of deprotonated ions at *m/z* 693.4

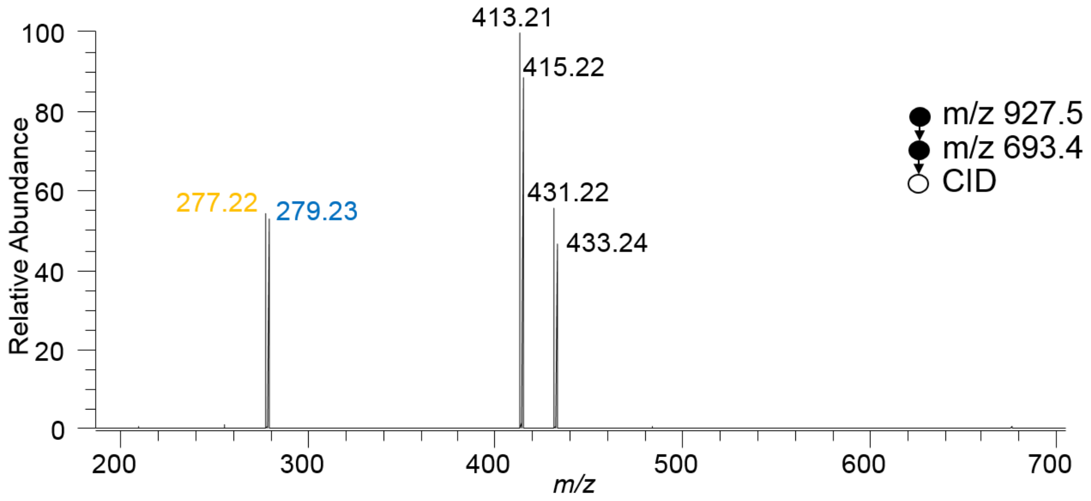

Figure S31. (a) MS^2 spectra of derivatized PA 36:5 at *m/z* 927.5 upon CID fragmentation (b) MS^3 spectra of deprotonated PA 36:5 at *m/z* 693.5 upon CID fragmentation. PA 36:5 can then be identified as PA 18:3 (Δ9, Δ12, Δ15)_18:2 (Δ9, Δ12).

Structural characterization of PA 37:3

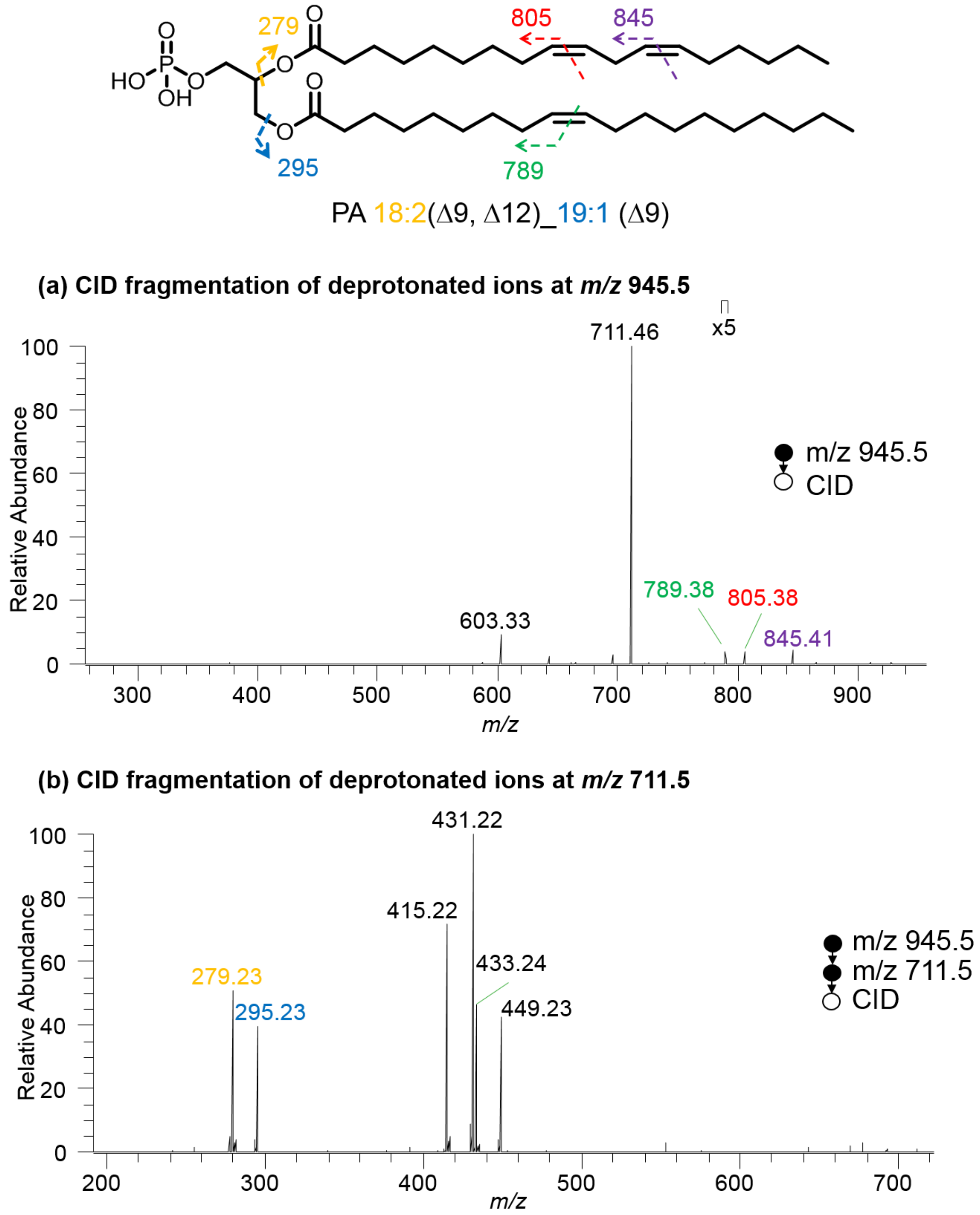

Figure S32. (a) MS^2 spectra of derivatized PA 37:3 at *m/z* 945.5 upon CID fragmentation (b) MS^3 spectra of deprotonated PA 37:3 at *m/z* 711.5 upon CID fragmentation. PA 37:3 can then be identified as PA 18:2 (Δ9, Δ12)_19:1 (Δ9).

Structural characterization of PA 37:4

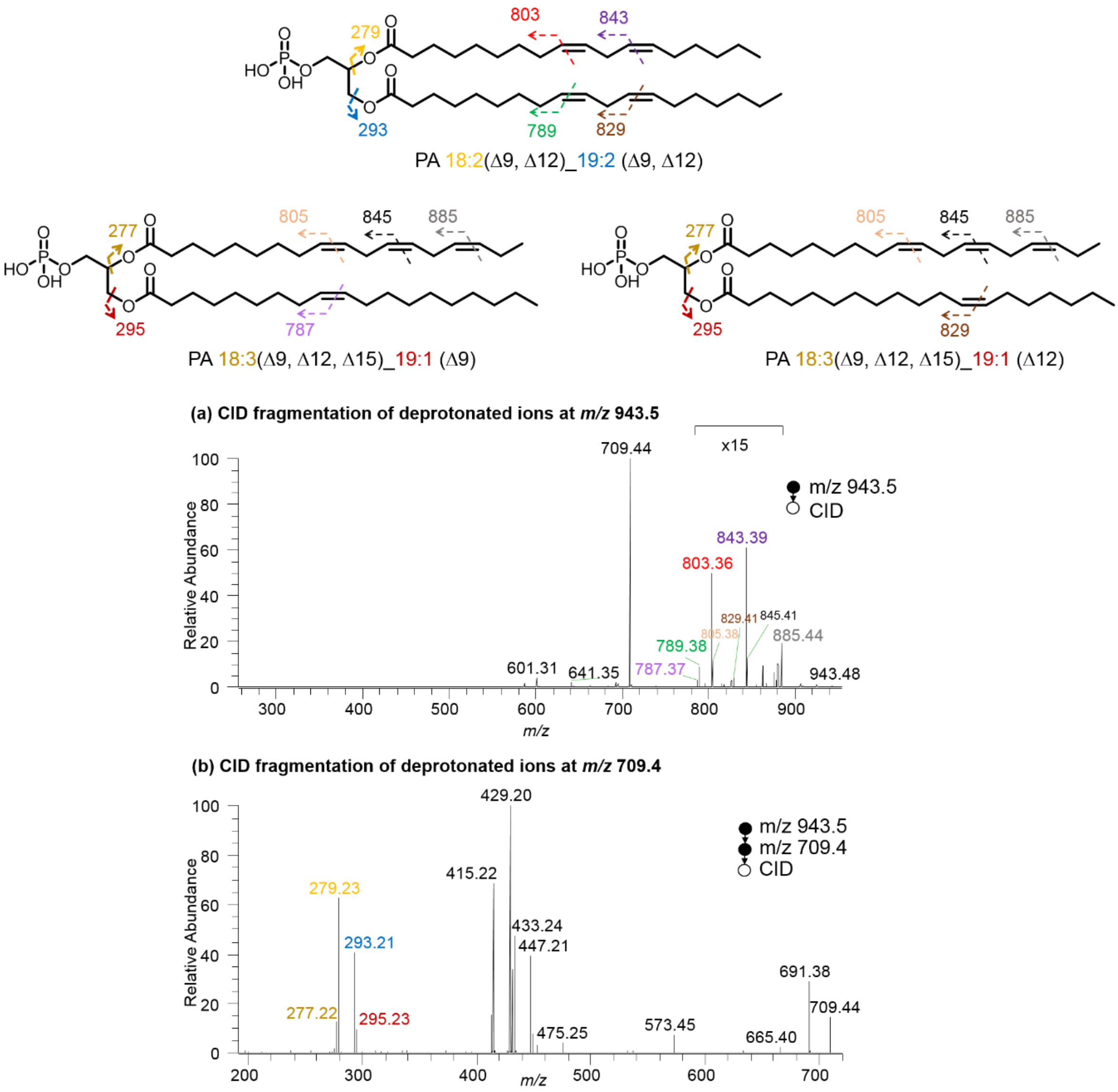

Figure S33. (a) MS^2 spectra of derivatized PA 37:4 at *m/z* 943.5 upon CID fragmentation (b) MS^3 spectra of deprotonated PA 37:4 at *m/z* 709.5 upon CID fragmentation. PA 37:4 can then be identified as PA 18:2 (Δ9, Δ12)_19:2 (Δ9, Δ12), PA 18:3 (Δ9, Δ12, Δ15)_19:1 (Δ9) and PA 18:3 (Δ9, Δ12, Δ15)_19:1 (Δ12)

Structural characterization of PG 34:1

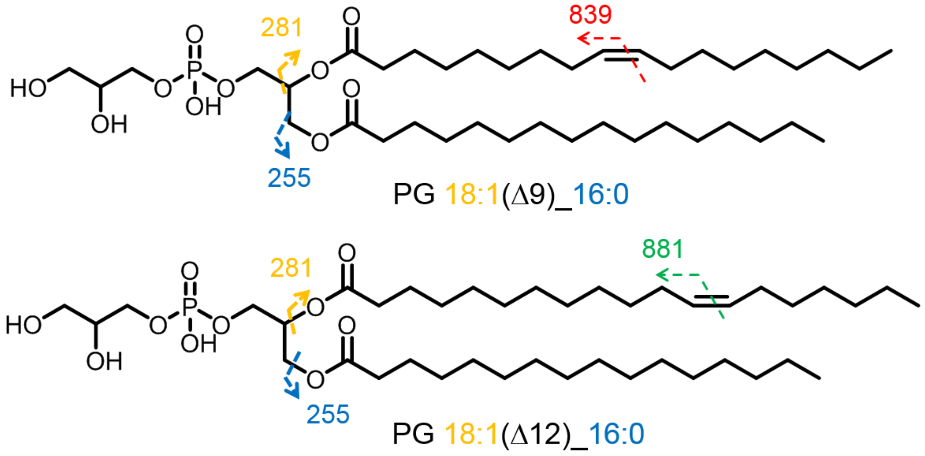

(a) CID fragmentation of deprotonated ions at *m/z* 981.5

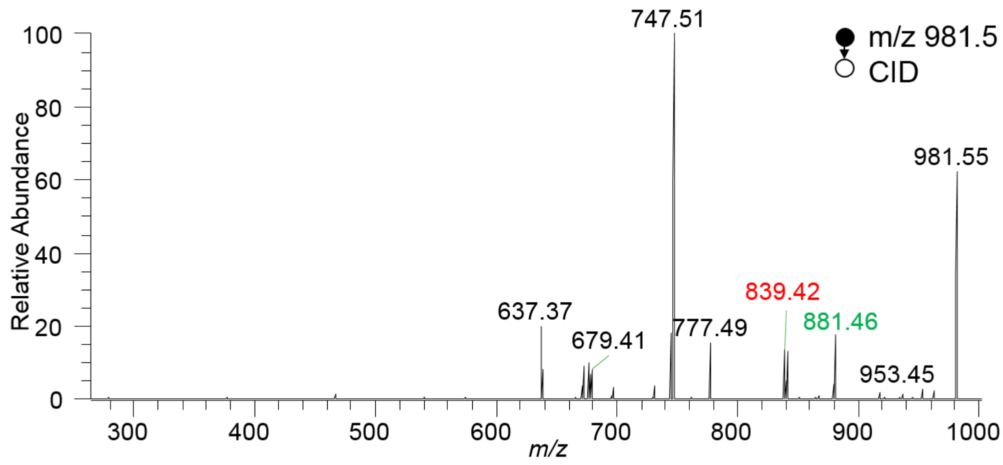

(b) CID fragmentation of deprotonated ions at *m/z* 747.5

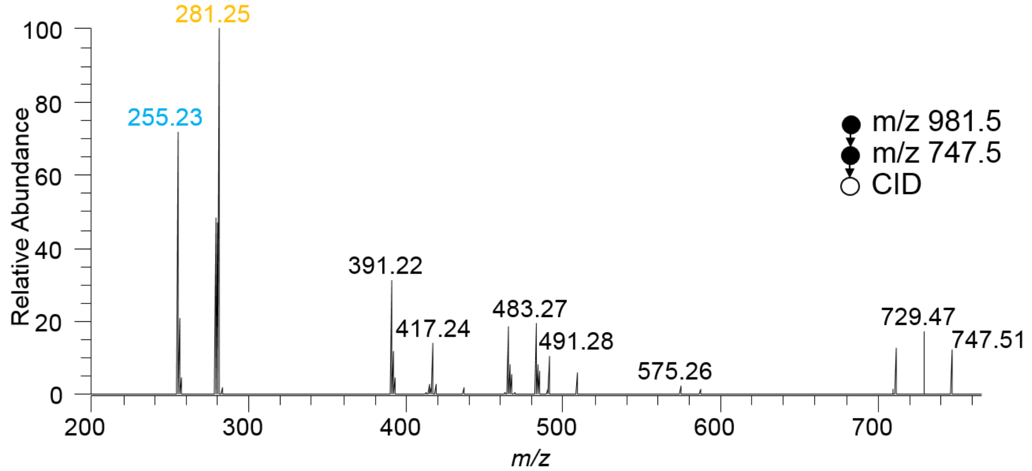

Figure S34. (a) MS^2 spectra of derivatized PG 34:1 at *m/z* 981.5 upon CID fragmentation (b) MS^3 spectra of deprotonated PG 34:1 at *m/z* 747.5 upon CID fragmentation. PG 34:1 can then be identified as PG 18:1 (Δ9)_16:0 and PG 18:1 (Δ12)_16:0.

Structural characterization of PG 34:2

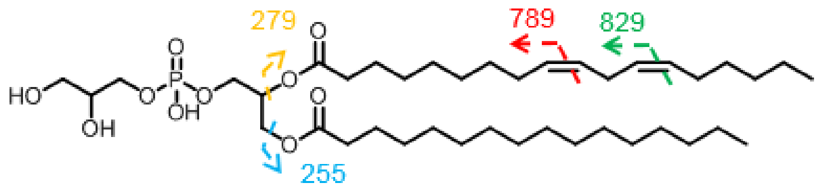

PG 16:0_18:2(Δ9, Δ12)

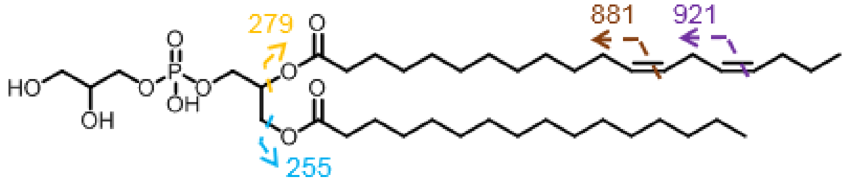

PG 16:0_18:2(Δ12, Δ15)

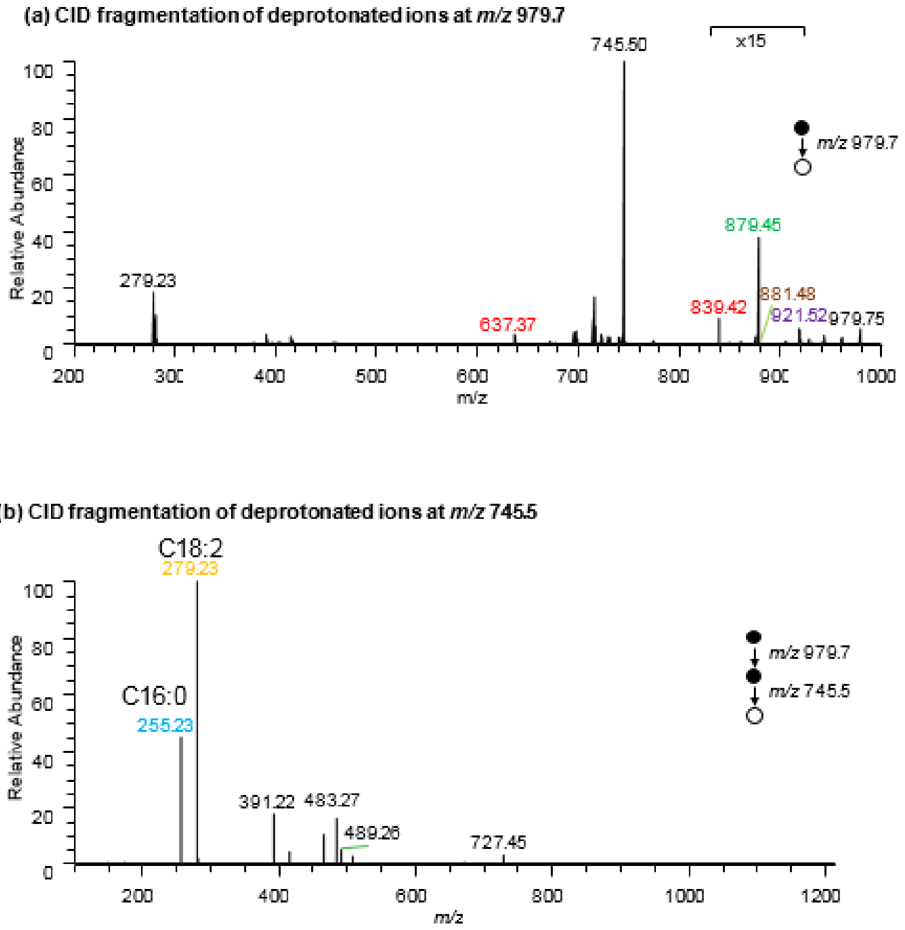

Figure S35. (a) MS^2 spectra of derivatized PG 34:2 at *m/z* 979.7 upon CID fragmentation (b) MS^3 spectra of deprotonated PG 34:2 at *m/z* 745.5 upon CID fragmentation. PG 34:2 can then be identified as PG 16:0_18:2 (Δ9, Δ12) and PG 16:0_18:2 (Δ12, Δ15).

Structural characterization of PG 35:1

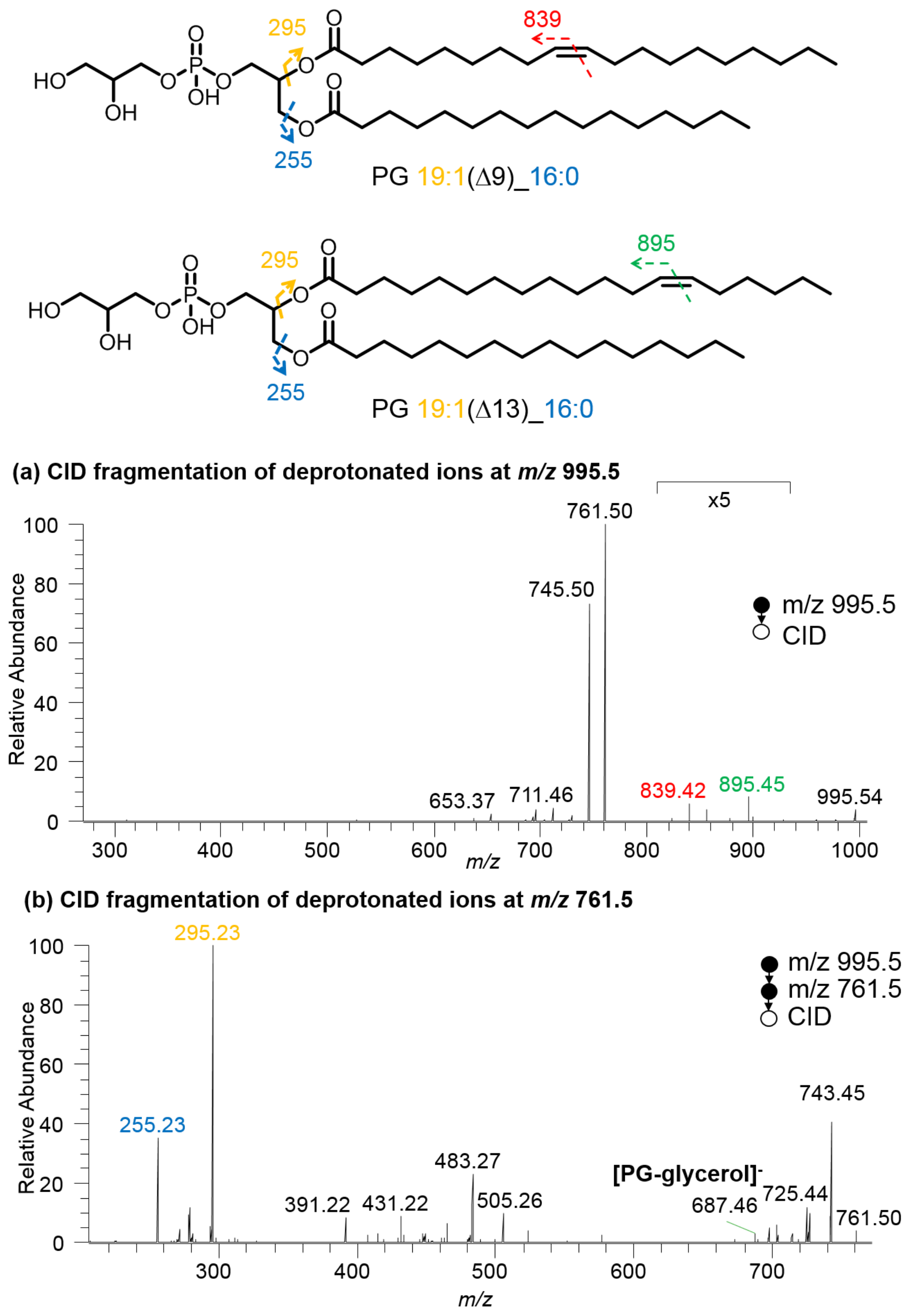

Figure S36. (a) MS^2 spectra of derivatized PG 35:1 at *m/z* 995.5 upon CID fragmentation (b) MS^3 spectra of deprotonated PG 35:1 at *m/z* 761.5 upon CID fragmentation. PG 35:1 can then be identified as PG 19:1(Δ9)_16:0 and PG 19:1(Δ13)_16:0

Structural characterization of PG 36:4

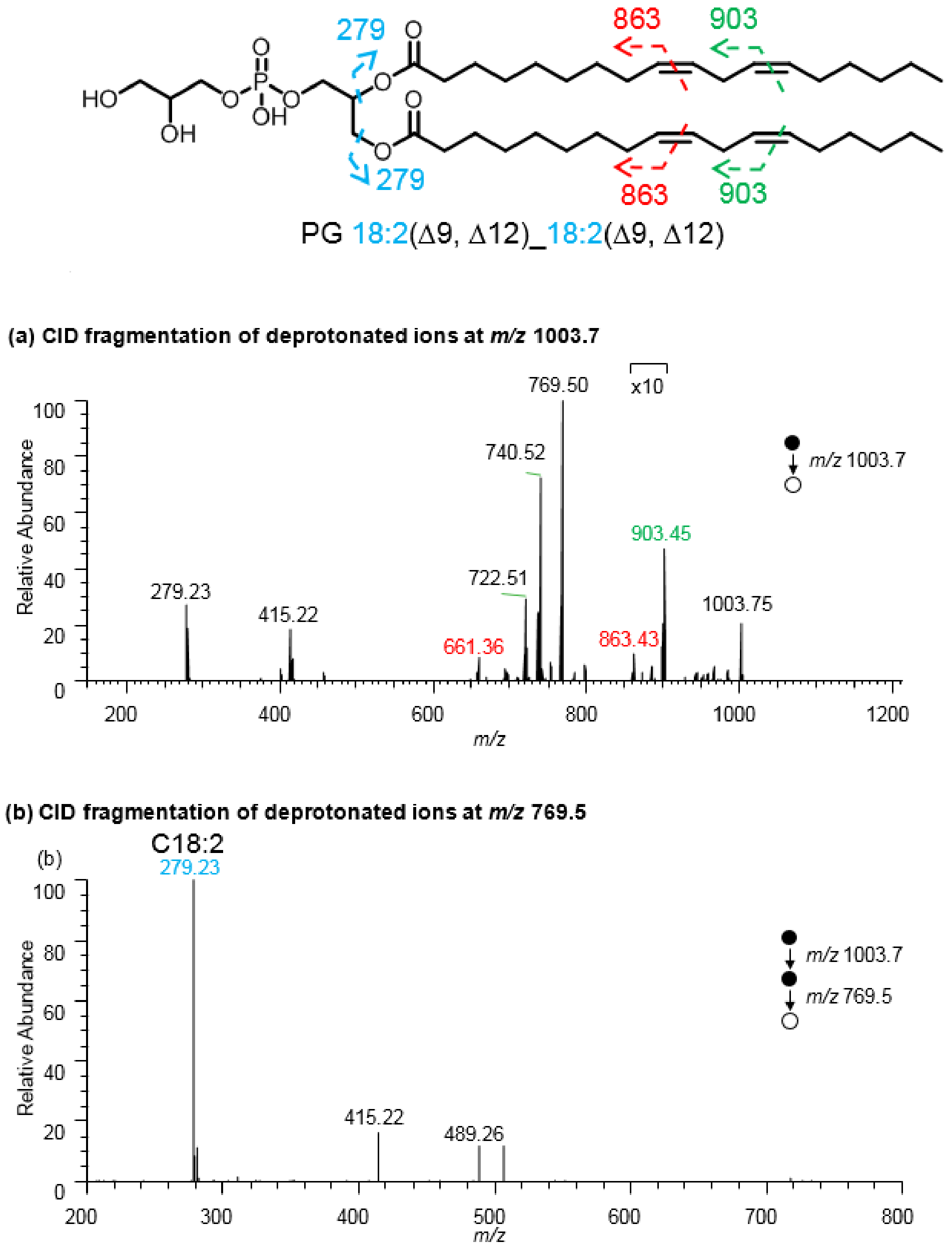

Figure S37. (a) MS^2 spectra of derivatized PG 36:4 at *m/z* 1003.7 upon CID fragmentation (b) MS^3 spectra of deprotonated PG 36:4 at *m/z* 769.5 upon CID fragmentation. PG 36:4 can then be identified as PG 18:2 (Δ9, Δ12)_18:2 (Δ9, Δ12).

Section S10. NMR spectra

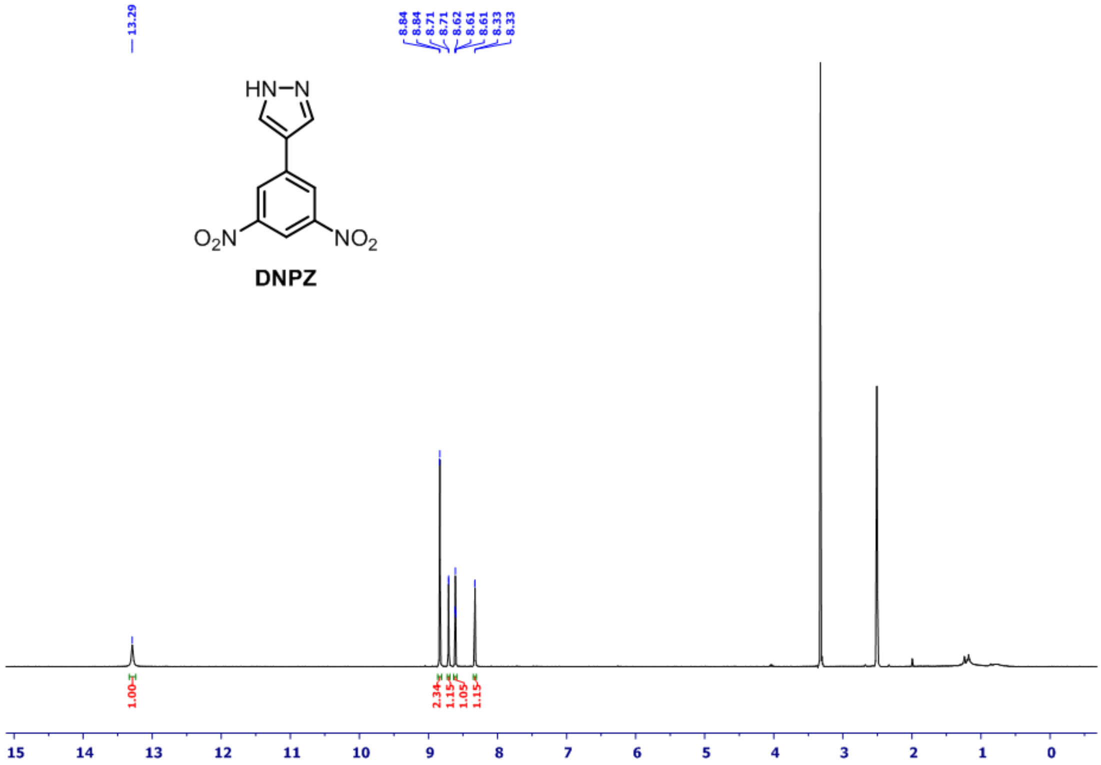

Figure S38. ^1H NMR spectra of dinitrophenyl pyrazole (400 MHz, DMSO-d_6)

References

1. Wise, D. E.; Gogarnoiu, E. S.; Duke, A. D.; Paolillo, J. M.; Vacala, T. L.; Hussain, W. A.; Parasram, M., Photoinduced Oxygen Transfer Using Nitroarenes for the Anaerobic Cleavage of Alkenes. *J. Am. Chem. Soc.* **2022,** *144*, 15437-15442.
2. Randolph, C. E.; Blanksby, S. J.; McLuckey, S. A., Enhancing detection and characterization of lipids using charge manipulation in electrospray ionization-tandem mass spectrometry. *Chem. Phys. Lipids* **2020,** *232*, 104970.
3. Stutzman, J. R.; Blanksby, S. J.; McLuckey, S. A., Gas-Phase Transformation of Phosphatidylcholine Cations to Structurally Informative Anions via Ion/Ion Chemistry. *Anal. Chem.* **2013,** *85*, 3752-3757.
